# Remnants from the Past: From an 18th Century Manuscript to 21st Century Ethnobotany in Valle Imagna (Bergamo, Italy)

**DOI:** 10.3390/plants12142748

**Published:** 2023-07-24

**Authors:** Fabrizia Milani, Martina Bottoni, Laura Bardelli, Lorenzo Colombo, Paola Sira Colombo, Piero Bruschi, Claudia Giuliani, Gelsomina Fico

**Affiliations:** 1 Department of Pharmaceutical Sciences, University of Milan, Via Mangiagalli 25, 20133 Milan, Italy; fabrizia.milani@unimi.it (F.M.); martina.bottoni@unimi.it (M.B.); laura.bardelli@studenti.unimi.it (L.B.); lorecolo.93@gmail.com (L.C.); pasico19@virgilio.it (P.S.C.); claudia.giuliani@unimi.it (C.G.); gelsomina.fico@unimi.it (G.F.); 2 Ghirardi Botanic Garden, Department of Pharmaceutical Sciences, University of Milan, Via Religione 25, 25088 Toscolano Maderno, Italy; 3 Department of Agricultural, Environmental, Food and Forestry Science and Technology, University of Florence, 50144 Florence, Italy

**Keywords:** ethnobotanical investigation, diachronic comparison, historical sources, medicinal plants

## Abstract

Background: This project originated from the study of an 18th century manuscript found in Valle Imagna (Bergamo, Italy) which contains 200 plant-based medicinal remedies. A first comparison with published books concerning 20th century folk medicine in the Valley led to the designing of an ethnobotanical investigation, aimed at making a thorough comparison between past and current phytotherapy knowledge in this territory. Methods: The field investigation was conducted through semi-structured interviews. All data collected was entered in a database and subsequently processed. A diachronic comparison between the field results, the manuscript, and a 20th century book was then performed. Results: A total of 109 interviews were conducted and the use of 103 medicinal plants, belonging to 46 families, was noted. A decrease in number of plant taxa and uses was observed over time, with only 42 taxa and 34 uses reported in the manuscript being currently known by the people of the valley. A thorough comparison with the remedies in the manuscript highlighted similar recipes for 12 species. Specifically, the use of agrimony in Valle Imagna for the treatment of deep wounds calls back to an ancient remedy against leg ulcers based on this species. Conclusions: The preliminary results of this study allow us to outline the partial passage through time fragments of ancient plant-based remedies once used in the investigated area.

## 1. Introduction

Diachronic approach in ethnobotanical studies has recently gained the attention of several authors. The use of historical sources allows researchers to collect valuable information about the mechanisms of transmission of Local Ecological Knowledge (LEK) and the nature of its transformation over time [1,2,3]. However, the interpretation of the results from diachronic studies is complicated by the intrinsic nature of LEK consisting of a complex multifaceted corpus of different practices, beliefs, and traditions having different origin and developmental patterns, molded by different socio-ecological factors. Some studies have shown that historical written records and iconography played a key role in shaping the modern Western medicinal knowledge as of the earliest manuscript copies of Dioscorides’ De Materia Medica. According to Leonti et al. (2009; 2010) [4,5], the long-lasting influence of Pietro Andrea Mattioli (1501–1577) on the medicinal plant uses in Southern Italy clearly transpires through the review of contemporary ethnobotanical literature. On the one side, the continuous use of some medicinal taxa through the millennia can be explained by their therapeutic efficacy supporting the concept of social validation [6]. However, different explanations, beyond the efficacy of the remedy, have been invoked to account for the maintenance, erosion, or reshaping of ethnobotanical knowledge in the modern cultural contexts. Among the factors identified: the abundance and the accessibility of plants [7], the influence of the educational systems [8,9], the role of urbanization processes [10], the impact of climate change on the distribution of medicinal species [11,12], the effects of economic and government policies on the flow of information [13,14], the role of media in popularization of plant uses [15,16], and the exchange between different cultures [6].

**Figure 1:**
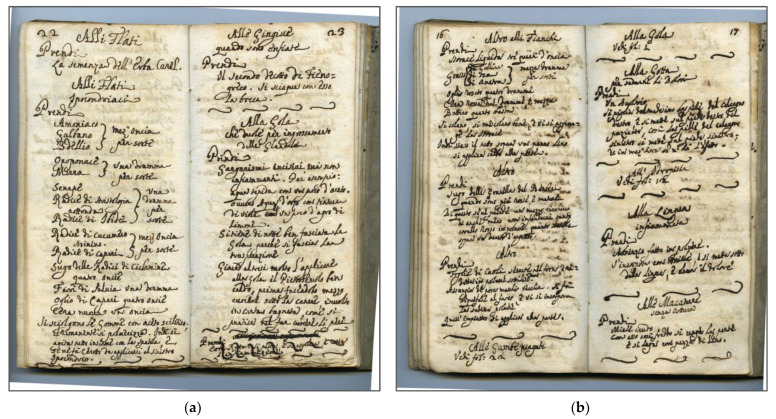
Pages taken from the 18th century manuscript: (**a**) remedies against oropharyngeal problems; (**b**) remedies against musculoskeletal and oropharyngeal disorders.

In 2021, Milani and Fico published a book titled ‘*Raccolta di diversi rimedj a varj mali*’ (Collection of various remedies to various pathologies), an in-depth ethnobotanical study of a pocket-size 18th century manuscript found in Valle Imagna (Bergamo, Lombardy, Italy) which contains approximately 200 plant-based complex recipes with therapeutic purposes collected and written down by an anonymous author [17] (Figure 1a,b). Some of these medicinal remedies had been retrieved from almanacs and medical textbooks and supplemented with personal notes from the author themselves. The manuscript was found in the private library of a 17th century house in Corna Imagna, one of the towns of the Valle Imagna (Bergamo, Italy), and was written in vulgar Italian with some references to Latin and to the vernacular tongue of Lombardy and the territory of Bergamo.

The remedies were considered useful to treat almost 80 different types of pathologies that were recognized by the official Western Medicine of that time (with the most treated pathologies being hemorrhoids, renal disorders, and ophthalmic problems) and were made of plant, mineral, animal, and human ingredients. Specifically, 205 plant species (21 of which are still unidentified) belonging to 70 botanical families were mentioned in the book, either used as they were or as derivatives. Today, most of these preparations can only be considered picturesque at best, because of the medical theories that the remedies were based on, the mix of ingredients used, the steps described for the preparations (which were often bound to religious rituals and superstitious beliefs), and the way of administering them to the patients. For example, in order to ‘*clear the eyes from the humors that cloud them’*, the author suggests to pierce the earlobes and put a piece of root of *Helleborus niger* L. in the holes as earrings; some of the preparations need to cook for ‘*as long as it is needed to run a Miserere*’; a preparation against kidney stones requires the collection of snails on a full moon night as the main ingredient [17]. However, bibliographic research in modern scientific literature showed that the use of some of the medicinal species involved and their active compounds could be potentially justified, to some extent, as treatment against some of the pathologies mentioned [17]. Admittedly, by the second half of the 19th century, improvements made in scientific research methodologies brought about the exclusion of ancient remedies, such as the ones described in the manuscript, from official Western Medicine standards. New pharmaceutical drugs (i.e., the first antibiotics, vaccines, semi-synthetic drugs, and others) contributed in no small part to this transformation [18]. However, in smaller and more isolated villages, such as the ones in Valle Imagna, this process was much slower and, even decades later, people frowned upon modern medicine, clinging to the ‘old ways’ instead, as reported by Dr. Giovanni Maconi MD in 2006 in his book ‘*La medicina popolare in Valle Imagna*’ (Folk medicine in Valle Imagna) [19]. As a family doctor, born at the beginning of the 20th century and raised in Valle Imagna, Maconi collected from family and personal experience, as well as from professional encounters and from some of the elderly of the valley, information regarding magical, ritualistic, and empirical components of folk medicine practices since early 20th century Valle Imagna, thus depicting a picture of the traditional knowledge of the territory at that time.

The results obtained from the analyses of the manuscript prompted us to deepen our investigation on traditional uses of spontaneous medicinal species still surviving nowadays in Valle Imagna, and on whether part of this knowledge could be potentially traced back to centuries past. Thus, we conducted an ethnobotanical survey to investigate current knowledge of medicinal plants in the upper Valle Imagna. Then, we performed a thorough comparison between the plants and their traditional uses currently known and possibly practiced by the people of the valley and the ancient remedies included in the 18th century manuscript described in Milani and Fico (2021). The book by Maconi (2006) was considered too, in the attempt to sketch a potential *fil rouge* across the centuries, from the 18th century medicine to the 21st century folk medicine of Valle Imagna.

## 2. Results and Discussion

### 2.1. Current Ethnobotanical Survey

Between November 2021 and November 2022, a total of 109 informants were interviewed in the field, 93 of whom provided information concerning the medicinal use of spontaneous and cultivated plants of Valle Imagna. Out of the total 3849 citations, 669 referred to the Medicinal sector and involved 103 plant species, belonging to 46 botanical families. Four of these species were recalled only by their vernacular name and the descriptions provided by the informants during the interviews were not sufficient to allow for prompt identification. For this purpose, further investigation, both on the field and bibliographic, is currently taking place.

Seventy-eight percent of the citations involved species collected in the wild, 17% cultivated species, and 1% species that were collected both spontaneous and cultivated (i.e., *Malva sylvestris* L., which is commonly found spontaneous in Valle Imagna, but was also sometimes grown by the informants in their own gardens). Finally, 5% of the citations referred to species that could not be found throughout the territory, but that were usually kept in the house and became common ingredients for herbal remedies (i.e., *Syzygium aromaticum* (L.) Merr. & L.M.Perry dried flower buds and *Zingiber officinale* Roscoe rhizomes).

The most recurrent botanical families were Malvaceae (n. citations = 188; n. species = 3), Asteraceae (100; 13), Lamiaceae (78; 7), and Rosaceae (68; 12), while the most cited species were *Malva sylvestris* (n. informants = 67; number of citations = 135), *Tilia cordata* Mill. (32; 52), *Taraxacum* sect. *Taraxacum* (21; 26), *Hypericum perforatum* L. (16; 27), *Sambucus nigra* L. (12; 20), *Laurus nobilis* L. (12; 19), *Rosa canina* L. (11; 15), *Agrimonia eupatoria* L. (10; 27), *Rosmarinus officinalis* L. (9; 14), and *Citrus* x *limon* (L.) Osbeck. fil. (9; 12). It can be observed that Malvaceae was the most mentioned family only due to the high number of citations concerning *M. sylvestris* and, to a lesser extent, *T. cordata*, while the citations for the others were distributed among a higher number of different plant species. It is interesting to highlight that during the preliminary bibliographic research conducted on the territory and the local flora [20,21,22,23], Asteraceae, Rosaceae, and Lamiaceae resulted also among the families with the higher number of spontaneous species distributed in the area with 27, 19, and 13 species, respectively. Ranunculaceae, with 19 species catalogued, was also the second most widespread family in Valle Imagna, although its species were rarely mentioned by the informants (only 5 out of the total citations), supposedly because of their common toxicity.

Concerning the 103 plant species mentioned by the informants, they were prepared mainly as infusions (n. citations = 340), used raw (i.e., externally applied as they were; n = 144), macerated in oil (n = 62), or boiled in decoctions (n = 48). Leaves (n. citations = 291; n. of species = 46) and flowers/inflorescences/flowered aerial parts (185; 26) were the most recurrent plant parts. Mostly, the reported uses were personally experienced (84.0% of citations) and still currently of concern (94.0%).

**Figure 2:**
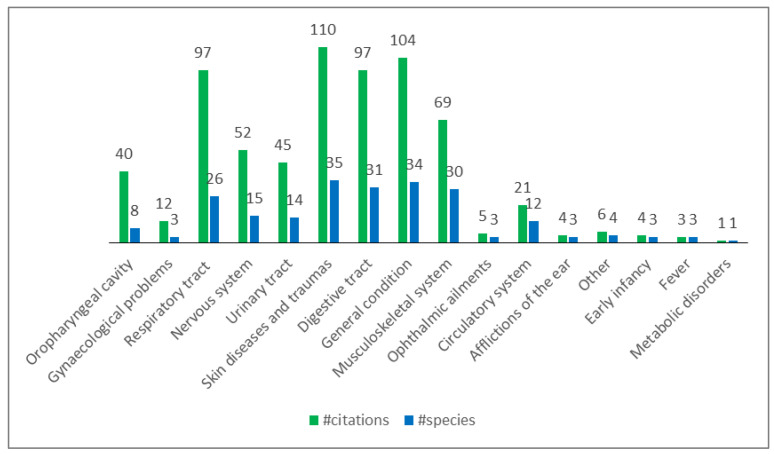
Categories of use (pathologies treated) in Valle Imagna. According to the number of citations (#citations) and number of species (#species).

The species were distributed among different categories of pathologies, according to their use (see Figure 2).

Among the 35 species used for the treatment of skin problems, flowers and aerial parts of *Hypericum perforatum* (n. citations = 21) were macerated for at least 3 weeks in vegetable oil. This oleolite was then applied on skin as an anti-inflammatory, wound healing, and soothing agent for wounds, sunburn, and burns. Compresses prepared with the leaves of *Malva sylvestris* (n = 11) were used mainly as anti-inflammatory and soothing for skin irritations. Leaves of *Agrimonia eupatoria* (n = 10) were applied fresh externally or as compresses of the infusion, mainly as a wound healing agent for wounds that were, as some of the informants reported, ‘hard to treat otherwise’.

In the category General condition, the infusion of leaves of *Malva sylvestris* (n = 38) was drunk for its general anti-inflammatory properties on the whole body, while the infusion of rosehips (*Rosa canina*, n = 8) was considered rich in vitamin C and useful as tonic and for the prevention of colds. The infusion of leaves and flowers of *Salvia officinalis* L. (n = 8) was drunk or added to the bath water as a tonic agent.

For the treatment of digestive problems, the infusion of leaves or flowers, or sometimes the whole above ground parts of *M. sylvestris* (n = 20) was drunk as laxative, or to ease abdominal pain. The leaves, rarely the flowerheads or the underground parts, of Taraxacum spp. (n = 16) were drunk in infusion or eaten mainly as liver depurative. The juice of *Citrus* x *limon* fruits (n = 7) was added to an infusion of *Thymus* spp., *Lavandula angustifolia* Mill., and *Zingiber officinale* to improve its digestive and antispastic properties. The juice was once added to *Ricinus communis* L. seeds oil and sugar as a laxative preparation. Finally, the infusion of leaves of *Artemisia absinthium* L. (n = 5) was taken for its digestive and carminative activities.

As for the category of respiratory tract infections, the most cited species was *Tilia cordata* (n = 30): its flowers were commonly collected and mainly used in infusions once dried for the treatment of cough, colds, and sore throat. Syrups of flowers (sometimes fruits) or infusions of flowers of *Sambucus nigra* (n = 13) were administered against cough for their expectorant properties, while a syrup of pinecones or fresh tops of *Pinus mugo* Turra (n = 8) was prepared for cough and sore throat. It is important to note that this last species can rarely be found throughout the territory of Valle Imagna and grows mainly at the higher altitudes of Mount Resegone (the highest mountain of the valley). For this reason, the informants who mentioned this use often collected the herbal ingredients for this preparation in the neighboring valleys, such as Val Taleggio and Val Brembana.

Among the 30 species used for musculoskeletal pains and inflammations, the most cited one was *Arnica montana* L. (n = 9). Its flowerheads were macerated in oil or in alcohol and the preparation used as it was or mixed with fat (i.e., bee wax) and rubbed externally on contusions or aching joints or muscles. For the same purpose, fresh leaves of *Brassica oleracea* L. (n = 7) were applied on the affected area. Moreover, the macerated oil of flowered aerial parts of *Achillea millefolium* L. (n = 5) was massaged (sometimes after mixing it with bee wax to obtain an ointment) on joints or on contusions and bruises.

The infusions of flowers of *T. cordata* (n = 11), flowered aerial parts of *L. angustifolia* (n = 8), and leaves of *Melissa officinalis* L. (n = 7) were the most mentioned preparations for the treatment of nervous system disorders, specifically as sedative remedies to facilitate and improve sleep. Additionally, the oleolite of lavender was massaged on the temples for the same purpose.

The most used species for the urinary tract was without doubt *M. sylvestris* (n = 21). The infusion of its leaves was drunk or used as vaginal douche for diuretic or anti-inflammatory purposes, specifically for cystitis and irritations. Another remedy against cystitis was the decoction of the whole above ground parts of *A. eupatoria* (n = 5), also drunk (in this case with the addition of cherry seeds) or used externally. The informants who reported this specific use of agrimony emphasized the fact that this remedy was once recommended by the midwives of the valley.

For the treatment of gingivitis and other inflammations of the oropharyngeal tract, the most used remedy was prepared once again with leaves (sometimes also flowers or underground parts) of *M. sylvestris* (n = 30). Its infusion was commonly used as anti-inflammatory mouthwashes. Less common was the similar use of the whole above ground parts of *A. eupatoria* (n = 2; decoction), the leaves of *Blitum bonus-henricus* (L.) Rchb. (n = 2; infusion), and the leaves of *Salvia officinalis* (n = 2; infusion or leaf applied on gums and teeth).

Concerning the circulatory system, leaves infusions of *Laurus nobilis* (n = 5) were considered useful mainly as a hypotensive, while the infusion of *Crataegus monogyna* Jacq. (n = 3) was drunk as a hypotensive as well, but also as an antiarrhythmic. Finally, decoctions of *Taraxacum* (n = 3) leaves or sometimes the leaves and underground parts eaten raw were considered a powerful blood depurative.

**Figure 3:**
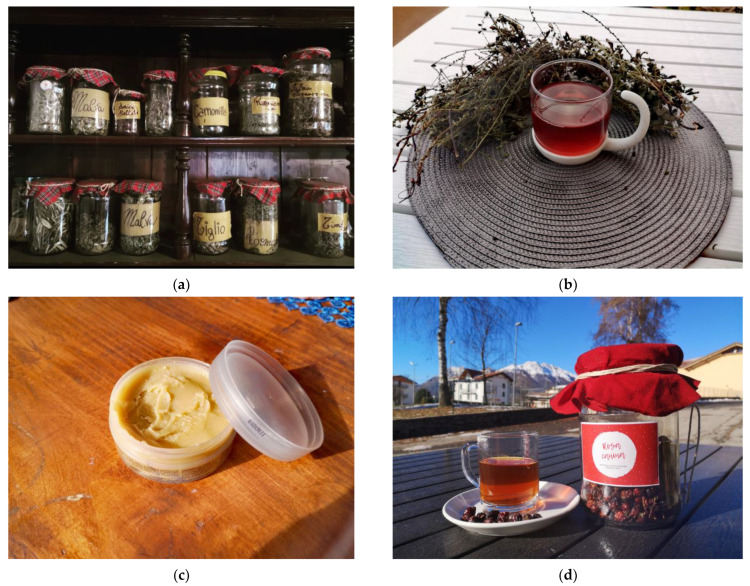
Some herbal preparations observed during the field work: (**a**) dried herbal teas of some of the most cited species, such as *M. sylvestris* and *T. cordata*; (**b**) decoction of aerial parts of *A. eupatoria*; (**c**) ointment obtained mixing extra virgin olive oil and bee wax; (**d**) decoction of rosehips (*R. canina*).

Figure 3a–d shows some of the preparations observed and collected during the fieldwork. For further information concerning the uses, please see Table S1 of the Supplementary Materials.

Finally, extensive bibliographic research in scientific literature was conducted on the 99 identified medicinal species mentioned by the informants in order to validate or refute their traditional uses. This bibliographic research highlighted that scientific evidence could be found for at least one of the uses concerning 62 taxa and detected during the field work, though it is important to note that the part of the plant and preparations analysed in literature rarely matched the traditional ones. Moreover, in vitro and in vivo studies could be more easily found, while clinical trials performed on human subjects were definitely lacking. Phytochemical investigations related to the potential biological activities were found only for 42 out of the 62 aforementioned taxa. For some of these, for example *Achillea millefolium*, *Calendula officinalis* L., *Malva sylvestris*, and *Sambucus nigra*, the literature concerning the active compounds involved in their activity and their potential mechanisms of action was relatively extensive, while in most cases the information was limited. The complete results of this research can be found in Table S1 of the Supplementary Materials.

Additionally, the analysis of knowledge distribution according to gender found that female and male informants presented different knowledge concerning medicinal plants, with females reporting a significant higher number of species (Mann–Whitney Test: U = 649; Z = −3.07; *p* < 0.01) and uses (Mann–Whitney Test: U = 703; Z = −2.64; *p* < 0.01) (Table 1). This finding was expected since women in Alpine areas represented the primary healthcare providers for the family until the recent past [8].

The age of informants ranged from 20 to 96 years with 70–79 years being the most frequent age class (32 informants) (Table 1). No significant correlation was observed either with the mean number of reported species (Spearman Test: R = −0.14) and the mean number of reported uses (Spearman Test: R = −0.11). Informants aged from 30 to 59 seem to be the most knowledgeable ones, with a mean number of mentioned species and uses of 5.18 (±0.51) and 7.88 (±1.63), respectively. At the same time, the 11 informants aged ≥ 80 presented the lower rates of knowledge (mean number of species: 3.11 ± 2.68; mean number of uses: 4.61 ± 4.42).

The same pattern was observed by Bruschi et al. (2019) [8] in an Alpine area close to Valle Imagna. We can interpret this pattern as a result of the cultural interest in medicinal plants by younger generations within the green wave and healthy lifestyle system. Part of the knowledge gathered from the younger informants came from several different sources other than the memories from their parents and grandparents. Some of them had recently rediscovered the wild plant species of their own territory through broad consultation of books, TV programs, and the Internet (i.e., a poultice of leaves *Delphinium consolida* L. for the treatment of fractures, or the macerated oil of *Hedera helix* L. leaves used to improve legs circulation against cellulitis). Others combined family reminiscences with new understanding gained through schools and university courses, upon deciding to return to their home territory with innovative agropastoral activities. An explanation of the low rate of knowledge owned by the older informants could be found in the history that distinguished the XIX century Valle Imagna. During the post World War II era, this valley was in fact characterized by sheer poverty, while its inhabitants were barely surviving through their rural lives with what the territory would offer [24]. From then on, many of them started looking for a better life by moving to neighbouring countries, mainly Switzerland and France, but also Germany; most of them never to return. Some of the people that came back years, sometimes even decades later, had lost most of their memories regarding that past farm life, thus leaving behind a great part of their knowledge on the traditional uses of the spontaneous plants of Valle Imagna. We personally interviewed some of these older people and we could determine first-hand that while they hardly recalled them, the uses that they were able to mention were also the ones most rooted to the territory and to the ancient traditions of the valley. As a way of example, we cite an ointment of *Allium sativum* L. underground organs and pork fat against contusions, the use of leaves of *Agrimonia eupatoria* externally applied fresh to treat deep wounds, or the aerial parts of agrimony drunk in anti-inflammatory infusions for the digestive and urinary tract. Additionally, it is interesting to note that these occurrences may have partly thwarted the intergenerational knowledge transfer of these traditional uses in Valle Imagna.

### 2.2. Diachronic Analysis

Before focusing on the plant species, it is important to note that some of the ancient non-herbal remedies described in the booklet and the ones from 20th centuries referred by Maconi are interestingly similar. Prolapsed hemorrhoids, for example, were usually treated with herbal or non-herbal ointments in order to slide them back and were then kept in position with a balled up linen cloth [17]. Maconi reports that until the early decades of the 20th century, children often suffered from rectal prolapse, which was treated with olive oil (with the same purpose of the ancient ointments) and that then the children were forced to sit naked on a cold stone, to keep the rectum in place [19]. Additionally, some of the ritualistic and religious elements that used to characterize the remedies from 18th century could also be found in the descriptions given by Maconi and from some of the stories recounted by our informants, especially the older ones. Religious formulas, such as fragments of prayers or the sign of the cross while taking or applying the remedies, were in some cases considered an essential part of them and were thought to enhance their power.

**Figure 4:**
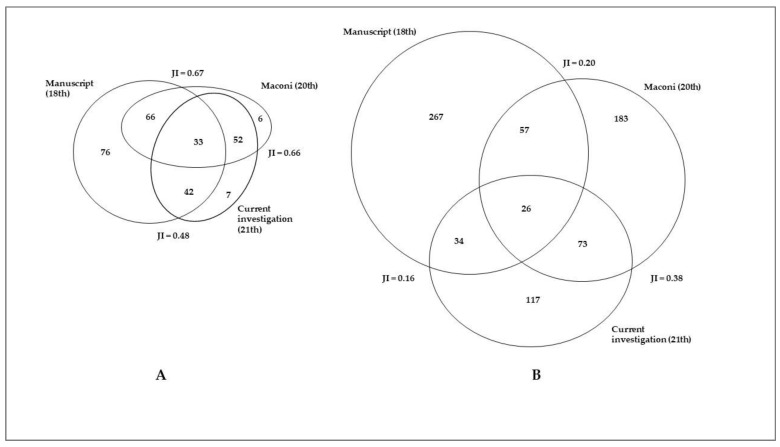
Venn diagrams comparing taxa (**A**) and uses (**B**) in the three data sources. JI = corrected Jaccard Index.

Figure 4 shows the comparison between the historical and current taxa and uses. A strong erosion in plant-related knowledge can be observed over time. Eighteenth century manuscript reported the highest number of taxa (184) and uses (358). These numbers are lower in Maconi’s book (125; 313) and in fieldwork (99; 224). The highest overlap was between the manuscript and the Maconi book for taxa (JI = 0.67) and between the current investigation and the Maconi book for uses (JI = 0.38) while the lowest one was between the manuscript and the current investigation (JI = 0.48 for taxa; JI = 0.16 for uses). Only 42 taxa (23% of the taxa reported in the manuscript) and 34 uses (9.5% of the uses reported in the manuscript) are currently known by the people living in the valley; in the 20th century investigation by Maconi, taxa and uses in common with those reported in the manuscript were 66 (35%) and 57 (16%), respectively.

In order to explain the observed differences between the manuscript and the other considered sources, it has to be highlighted that the manuscript includes remedies taken from other sources, even from earlier times to 18th century, not always connected with the territory of Valle Imagna. For example, among these “external” sources identified by Milani and Fico (2021), “*Prospectus Phamaceuticus sub quo Antidotario Mediolanense*”, published in 1668 and updated until 1729; “*Pratica universale nella Medicina*”, dated 1693 and written by friar Felice Passera from Bergamo; and “*De Secreti del Reverendo Donno Alessio Piemontese*”, dated 1557 and written by Girolamo Ruscelli. Even the high number of exotic species reported in the manuscript (54, 14 of which neophytes) compared to Maconi (29; 8) and the fieldwork (17; 7) can further explain the observed differences. Since the 16th to 18th century, Bergamo was part of the “*Repubblica Serenissima di Venezia*”, one of the most important centers of trade in Europe at that time. For this reason, doctors and apothecaries could obtain a wide variety of spices and exotic ingredients.

The comparison between Maconi’s book and the results of our fieldwork show that in a few decades, information on 73 taxa (58.4%) and on 240 uses (77%) was lost. A rapid loss of ethnomedicinal knowledge has been detected in many industrialized countries [8,25,26] and has been explained as a consequence of the cultural erosion caused by the influence of modern culture and education systems, the globalization of trade, and access to modern medicine [25].

**Figure 5:**
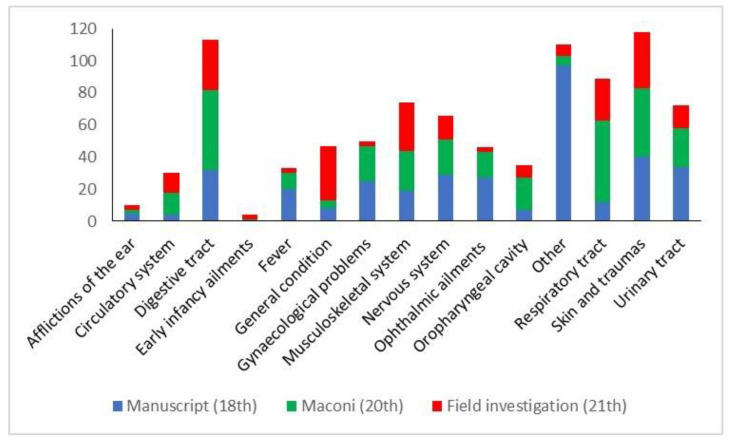
Distribution of the use categories in the three data sources.

Taking into consideration the categories of use, skin and digestive tract diseases are the ones with the highest number of taxa with a homogeneous distribution in the three datasets (Figure 5).

Regarding the manuscript, “others” is the most represented category with 97 recorded taxa; it mostly includes diseases or symptoms which are not easily interpretable and classifiable in accordance with Western nosologies (i.e., ’*ponta*’, ‘*brossole*’, ‘*tarlo*’, which cannot be translated). Taxa reported in all the three data sources were found in the following categories: skin diseases and traumas (10 taxa: *Chelidonium majus* L., *Citrus* x *limon*, *Malva sylvestris*, *Matricaria chamomilla* L., *Olea europaea* L., *Plantago major* L., *Plantago* ssp., *Salvia officinalis*, *Sambucus nigra*, *Urtica* spp.), respiratory tract diseases (5: *Linum usitatissimum* L., *M. sylvestris*, *Plantago* spp., *Rosa canina*, *Rosmarinus officinalis*), digestive tract diseases (2: *Artemisia absinthium*, *R. officinalis*), circulatory system disorders (1: *Urtica* spp.), urinary tract diseases (1: *M. syslvestris*), nervous system diseases (1: *M. chamomilla*), gynaecological disorders, obstetric, and puerperal problems (1: *M. sylvestris*), general condition (1: *S. officinalis*). A constant decrease in the number of the recorded taxa over time can be observed in the categories fever (20:10:3), gynaecological disorders, obstetric, and puerperal problems (25:22:3), nervous system diseases (29:22:15), ophthalmic ailments (27:16:3), and urinary tract diseases (34:24:14). Skin diseases (40:43:35) and digestive tract diseases (30:50:31) categories showed a more heterogeneous pattern but always with a reduction in the more recent years. Similar findings were observed by Söukand et al. (2022) [27] in Estonia and by Guarrera in Italy (2006) [28]. On the contrary, an increase can be detected for musculoskeletal disorders (19:25:30), circulatory system disorders (4:14:12), and general condition (8:5:34). As pointed out by Dal Cero et al. (2023) [6], the increasing importance of plants for treating circulatory system disorders and plants used as preventive measures, like plants administrated as blood and organs purifier or appetite stimulant/tonic, reflect the progress of medical knowledge and also epidemiological changes occurring in modern society [27].

**Figure 6:**
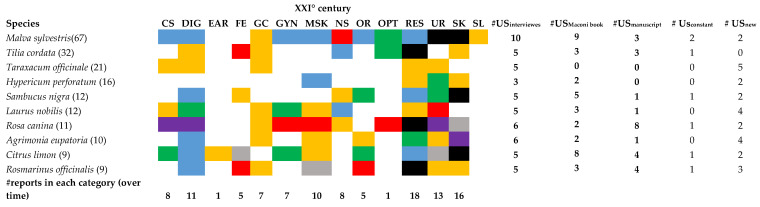
Categories of use for the 10 most cited species in the field investigation: comparison between the three data sources. CS = Circulatory system disorders; DIG = Digestive tract disorders; EAR = Afflictions of the ear; FE = Fever; GC = General condition; GYN = Gynaecological disorders, obstetric, and puerperal problems; MSK = Musculoskeletal system disorders and traumas; NS = Nervous system disorders; OR = Oropharyngeal cavity affections; OPT = Ophthalmic ailments; RES = Respiratory tract infections; UR = Urinary tract disorders; SK = Skin diseases and traumas; SL = Slimming. In brackets: number of informants citing the species. Black: the use has been cited during the interviews and is reported in the two books; orange: the use has been cited during the interviews but is not present in the two books; blue: the use has been cited during the interviews and is reported in Maconi; violet: the use has been cited during the interviews and is reported in the manuscript; green: the use is reported only in Maconi; red: the use is reported only in the manuscript; grey: the use is reported both in Maconi and the manuscript. #UScostant = number of uses surviving through the time; #USnew = number of uses recently appeared.

Figure 6 shows the uses categories of the 10 most cited species during the interviews compared to those reported in the two historical sources. Only in 6% of the cases (*M. sylvestris* used to treat skin problems and urinary tract diseases; *T. cordata* used to treat respiratory tract diseases; *S. nigra* used to treat skin problems; *R. canina* used to treat respiratory tract diseases; *Citrus* x *limon* used to treat skin problems; *R. officinalis* used to treat respiratory tract diseases), the use was the same in the three sources. Fourteen percent of the uses were reported only in Maconi and in the fieldwork. Twenty-seven percent were new (i.e., were cited by the informants during the interviews but they were not reported in the two historical sources). In particular, new uses were recorded in the category general condition, a finding further confirming the increasing importance of preventive plants in the current herbal medicine. Although the use of *Taraxacum* as remedy to treat digestive, urinary, respiratory diseases, and as a blood purifier has been known since ancient times [29,30], no records were found in the two historical sources analysed in this study. This finding can partly be explained by possible identification problems of species included in the 18th century manuscript; on the other hand, no information about medicinal uses of Taraxacum spp. over the last 200 years were reported in Dal Cero et al. (2023) [6] for Central Europe and Dal Cero et al. (2014) [31] for Switzerland. In general, these data are consistent with what was reported by other diachronic studies. For example, when comparing the uses of *M. sylvestris*, *H. perforatum*, *S. nigra*, *R. canina*, and *A. eupatoria* to those reported in Dal Cero et al. (2023) for the same species, we found a Jaccard similarity index of 0.72.

### 2.3. Comparison among Similar Preparations in 18th and 21st Century in Valle Imagna

Out of the 42 species shared between the manuscript and our field investigation, 12 were used in remedies that were at least comparable, sometimes even almost identical. All information regarding the comparison can be found in Table 2.

More specifically, for 5 species (*Chelidonium majus*, *Linum usitatissimum*, *Plantago* spp., *Salvia officinalis*, and *Vitis vinifera*) the 18th century remedies and the traditional uses in Valle Imagna were similar not only in terms of part of the plant used, but also of methods of preparation and administration, and the same uses were reported by Maconi in his book.

For *V. vinifera*, byproducts of the species were used, namely wine or grappa. Wine was one of the mediums in 18th century in which plants were boiled to obtain disinfectant mouthwashes in case of gingivitis, while nowadays in Valle Imagna another byproduct of *V. vinifera*, grappa, could be used pure for the same purpose. Admittedly, in this specific case, the disinfectant action is probably sought after in the alcoholic nature of the preparations, as well as in the plant species infused. As a matter of fact, the author described a preparation of leaves of sage and rosemary boiled in wine with pomegranate used as mouthwashes against painful and loosening teeth. Our informants reported the use of sage leaves rubbed directly on teeth and gums as an anti-inflammatory remedy and of a sage infusion used as anti-inflammatory and disinfectant mouthwashes for teeth and gums. It is interesting to highlight that Maconi recounted in his book that, until the first half of 20th century, mouthwashes obtained by boiling sage in wine were a typical treatment for gum and tooth ache in the valley [19].

A similar consistency with the data reported by Maconi could also be found in the cases of *C. majus*, *L. usitatissimum*, and *Plantago* spp. As a matter of fact, the orange latex obtained by snapping the stems of celandine was mentioned as a useful treatment for warts in all three of the consulted sources. In a similar fashion, a warm poultice of boiled flaxseed was externally applied on the chest to treat productive cough and was reported by both Maconi and our informants with the same vernacular name (‘*linusa*’), while in 18th century the mucilaginous water obtained by boiling the flaxseeds was added to fresh butter and then applied on the chest, specifically in children. As for *Plantago* spp., our informants, in accordance with what was written by Maconi, reported the use of leaves of plantain applied externally on burns and as a wound healing and anti-inflammatory remedy. For similar purposes, the author of the manuscript described the preparation of two different ointments, both obtained by mixing a poultice of plantain leaves with butter and other ingredients.

At last, an interesting case is represented by *Agrimonia eupatoria*. In the manuscript, the author referred to a complex remedy to treat leg ulcers. Specifically, they wrote: “*Alle Piaghe: D’ogni sorte nelle Gambe, sebbene la Gamba fosse scoperta e mangiata fino all’osso*” (To [treat] any type of leg ulcers, even if the leg was open raw and consumed to the bone) [17]. This remedy was prepared by boiling above ground parts of agrimony and dried roses in wine, which was then dabbed on the leg to disinfect the ulcers. A mix of powdered herbal and mineral ingredients was then applied on the wounds. Finally, the sediment of boiled agrimony and rose was smeared over the powder, and all was set in place with a gauze. While recounting the use of agrimony in Valle Imagna, our informants spoke about the external application of fresh leaves of the plant on deep wounds, which were considered ‘untreatable with any other method’. The leaf was then set in place with a gauze that was periodically changed. Moreover, they also described the use of compresses of the leaves or infusion of above ground parts, for the treatment of deep wounds as well. It is interesting to note that the use of agrimony was mentioned almost exclusively by elderly people (75 years or older) and that the plant species was only referred to by its vernacular name, which is ‘*Erba del Vinil*’, as not even the Italian common name was known to them. Furthermore, the primary data of our field investigation revealed that *A. eupatoria* was used in this fashion mainly until the 1960s–1970s, coinciding with the great migration of people from rural areas to the big cities and foreign countries. Finally, extensive bibliographic research conducted on ethnobotanical published works on neighboring areas has shown that in Lombardy, *A. eupatoria* was either used to treat different pathologies (Vitalini et al., 2009–infusion drunk for laryngitis [32]; Vitalini et al., 2015–compresses of the infusion for mild dermatitis and infusion drunk as an astringent agent, [33]), or not mentioned at all [8,34,35,36,37].

## 3. Materials and Methods

### 3.1. Area of Investigation

**Figure 7:**
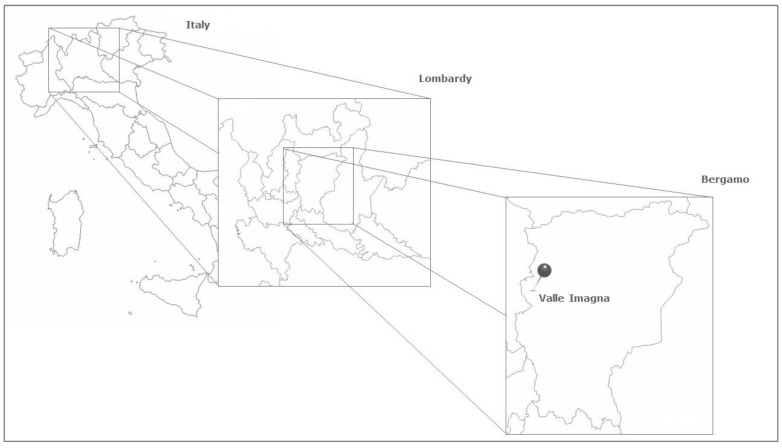
Valle Imagna is located in the province of Bergamo, Lombardy region, Northern Italy. Map modified from the original free maps at https://d-maps.com/carte.php?num_car=284951&lang=it (accessed on 14 July 2023), https://d-maps.com/carte.php?num_car=22383&lang=it (accessed on 14 July 2023), and https://d-maps.com/carte.php?num_car=213806&lang=it, (accessed on 14 July 2023).

Valle Imagna is located in the western area of Orobic Prealps, in the province of Bergamo, Lombardy, Northern Italy (Figure 7). The highest peak of the Valley is mount Resegone, with its 1.875 m a.s.l. Although some of its municipalities do not exceed 500 m a.s.l., the whole territory is labelled as mountainous. The main river of the area, called Imagna, gives its name to the Valley. The mild weather, with its abundant rainfalls and limited temperature excursion, allows for the presence of rich plant biodiversity. The woodlands are characterized almost exclusively by deciduous trees. Specifically, at the higher altitudes, beech woods are so extensive that several toponyms derive from the beech Italian common name, ‘*faggio*’ and the vernacular name ‘*fó*’. Concerning herbaceous plant species, among the most abundant we cite *Achillea millefolium* L., *Agrimonia eupatoria* L., *Arctium lappa* L., *Artemisia* L. spp, *Carum carvi* L., *Chelidonium majus* L., *Cichorium intybus* L., *Equisetum arvense* L., *Polypodium vulgare* L., *Filipendula ulmaria* (L.) Maxim., *Foeniculum vulgare* Mill., *Gentiana* L. spp, *Hedera helix* L., *Hypericum perforatum* L., *Malva sylvestris* L., *Ruta graveolens* L., *Salvia* L. spp, *Urtica dioica* L., and *Vaccinium myrtillus* L. [19]

For our investigation, we focused on upper Valle Imagna, specifically on the municipalities of Berbenno (675 m a.s.l.), Brumano (911 m a.s.l.), Corna Imagna (736 m a.s.l.), Costa Valle Imagna (1.014 m a.s.l.), Fuipiano Valle Imagna (1.055 m a.s.l.), Rota d’Imagna (690 m a.s.l.), and Sant’Omobono Terme (427 m a.s.l.).

### 3.2. Ethnobotanical Survey, Data Archiving and Processing

In preparation of the field work, preliminary bibliographic research on the territory and the spontaneous flora of Valle Imagna was performed through the consultation of local botany textbooks [20,21,22,23]. A list of autochthonous species of the Valley was produced. The scientific names and family categorization follow Pignatti et al., 2017 [38]. The list was then enriched by a photographic archive of the plants, in order to facilitate plant identification by the informants during the field work.

Subsequently, open- and semi-structured interviews were conducted. All the information gathered during the interviews was archived in Microsoft Word^TM^ files (Microsoft, Redmond, WA, USA): an ‘Informant Sheet’ for data concerning the informants and a ‘Species Sheet’ for data concerning the plant species cited and their traditional uses throughout the Valley. Specifically, the “Species Sheet” consisted of a 7-column table arranged as follows: Species (common and vernacular name), Field of use, Detailed use, Preparation form, Administration form, Part of the plant, Other information. Each “Informant Sheet” was then matched to the corresponding “Species Sheet” through a one-to-one identification code. Identification of the species was performed by Professor Gelsomina Fico, Professor Claudia Giuliani, and Dr. Paola Sira Colombo following Pignatti et al., 2017 [38]. Plant species nomenclature was according to Pignatti et al., 2017 for the ones found on Italian territory and to http://www.worldfloraonline.org/ (accessed on 14 July 2023) [39] for the other ones.

### 3.3. Data Analysis

All data was filed away in a database, organized in an Excel^TM^ spreadsheet (Microsoft, Redmond, WA, USA) where each row represents a ‘citation’, defined as ‘a single use reported for a single species by a single informant’. Every citation was considered as ‘distinct’, when differing from one another in at least one of the following fields: species, informant id code, category of use, part of the plant, preparation, and administration form. Data was processed and analysed by means of Pivot tables.

Spearman’s correlation test was carried out to detect a relationship between the age of informants and their plant-related knowledge. Mann–Whitney was performed to check for statistical significance between sex of informants and their ethnobotanical knowledge. To measure the similarity of the different data sources in terms of reported taxa and uses, we performed the Jaccard Similarity Index (corrected for maximum possible values, as reported in Pitman et al. (2005), in order to account for artefacts due to the difference in diversity between the considered data sources) [40].

For the comparisons among the different sources, we defined ‘use’ as ‘category of use’, namely the affected organ groups, such as ‘digestive tract disorders’, ‘urinary tract disorders’, etc. The followed criterion for the classification of the different categories of use was based on previous published works [34,35,41,42,43].

### 3.4. Bibliographic Research

For the comparison between the 18th and 20th century remedies and the 21st century ones, we consulted Milani and Fico, 2021 [17] and Maconi, 2006 [19], respectively. Comparing information in diachronic studies is complicated by the strong heterogeneity existing among the different sources [16]. Different authors employ different methodological approaches to record, systemize and present data, apply different categories and terminologies to describe different plant uses, often without any annotation, and use different plant nomenclatures. We used a rigorous approach to achieve data harmonization, punctually identifying, removing, and aligning all the study differences; this could have led to a loss of information but also provided a comparable view of data from different sources and revealed valid inferences from the analysis of pooled data.

Extensive bibliographic research in the ethnobotanical, phytochemical, and concerning the biological activities of the 99 identified species mentioned during the interviews was carried out through search engines and online databases, such as PubMed, MEDLINE, Google Scholar, and the bibliographic research online tool known as J.A.N.E. Concerning the ethnobotanical research, the scientific or English common name of the species was combined with the keywords ‘ethnobotany’, ‘ethnopharmacology’, ‘traditional medicine’, or ‘folk medicine’. Special attention was paid to Italian ethnobotanical works published and, specifically, the ones concerning the neighboring areas to Valle Imagna. As for phytochemistry and biological activities, we paired the scientific or English common name with specific keywords concerning the category of use cited in Valle Imagna (i.e., *Artemisia absinthium* AND digestive system, *Agrimonia eupatoria* AND skin) and then the specific pathology or activity (i.e., *Artemisia absinthium* AND carminative, *Agrimonia eupatoria* AND wound healing, etc.). We focused our attention particularly on systematic reviews and meta-analyses, if possible, or on single in vitro, in vivo, and clinical trials studies, and we applied no time filters. Table S1 of Supplementary Materials was then produced [32,33,34,35,36,37,44,45,46,47,48,49,50,51,52,53,54,55,56,57,58,59,60,61,62,63,64,65,66,67,68,69,70,71,72,73,74,75,76,77,78,79,80,81,82,83,84,85,86,87,88,89,90,91,92,93,94,95,96,97,98,99,100,101,102,103,104,105,106,107,108,109,110,111,112,113,114,115,116,117,118,119,120,121,122,123,124,125,126,127,128,129,130,131,132,133,134,135,136,137,138,139,140,141,142,143,144,145,146,147,148,149,150,151,152,153,154,155,156,157,158,159,160,161,162,163,164,165,166,167,168,169,170,171,172,173,174,175,176,177,178,179,180,181,182,183,184,185,186,187,188,189,190,191,192,193,194,195,196,197,198,199,200,201,202,203,204,205,206,207,208,209,210,211,212,213,214,215,216,217,218,219,220,221,222,223,224,225,226,227,228,229,230,231,232,233,234,235,236,237,238,239,240,241,242,243,244,245].

## 4. Conclusions

Our previous analysis of a little 18th century manuscript found in Valle Imagna was the starting point of the case study reported in this paper. The library where the manuscript was retrieved was part of a private 17th century house, stocked with historical documents related to the valley, and the presence in the text of vernacular words from the province of Bergamo could be indication of the provenance of the author. As for the purpose of such a piece, the personal and professional notes that the anonymous author added to various remedies would suggest that the work was considered more than a mere copy from other sources. Even the size of the booklet hinted at its practicality to be carried around and produced when a consultation was needed, maybe even by a medical practitioner of the territory.

The first comparison with published books concerning 20th centuries folk medicine in the valley prompted us to deepen our understanding of the traditional knowledge still surviving nowadays in Valle Imagna, specifically on the uses of plant species. Our survey revealed that, especially after the 1960s and 1970s great migrations from the valley, this knowledge was almost completely lost to the passage of time and the fading of memories. However, a few older people of the valley could still recall some of the traditional uses of spontaneous plant species and being able to retrieve this information and partly stop the loss of memories was certainly an important step.

The diachronic comparison among the historical sources involved in this analysis confirmed a general pattern of decline in the information concerning both number of taxa and their uses across the centuries, from the 1700s, represented by the manuscript, through the early 1900s, described by Maconi, to the first two decades of the 2000s, highlighted by our field investigation. Another clear example of the pending loss of knowledge concerns the uses of the first 10 frequently mentioned species during the fieldwork, compared to the ones of the historical sources.

However, among the similar uses, the 18th and 21st century preparations regarding *Agrimonia eupatoria* undoubtedly piqued our interest, due not only to the shared purpose of use and desired outcome of the remedies, but also to some similarities concerning the part of the plant used and their administration form. Moreover, although some of the other species are commonly used throughout Lombardy, the bibliographic investigation concerning the ethnobotanical studies conducted in neighboring areas underlined the specific and identifiable use of agrimony in Valle Imagna.

Further investigation is certainly mandatory but ultimately, our analysis of the sources allowed us to likely sketch a *fil rouge* across centuries of traditional knowledge of Valle Imagna, with unfortunately only slivers of the ancient remedies of the 18th century surviving the passage of time.

## Figures and Tables

**Table 1:** Mean number (±sd) of species and uses according to the gender and age classes of informants.

Gender	#Informants	Species		Citations	
		Mean	±(sd)	Mean	±(sd)
Male	37	3.57	4.65	5.51	8.21
Female	56	5.25	4.67	8.44	9.29
**Age**					
20–29	5	4.30	7.30	9.37	5.90
30–39	6	4.66	5.31	6.16	7.65
40–49	10	5.30	3.79	8.10	7.40
50–59	16	5.69	7.31	9.37	10.36
60–69	13	4.46	2.29	8.46	6.60
70–79	32	4.40	4.99	6.62	8.70
80–89	9	3.22	2.43	5.22	6.02
90–96	2	3.00	2.83	4.00	2.83
**All informants**	93	4.58	4.70	7.28	8.95

**Table 2:** Comparison among similar preparations found in the manuscript [17] and during the field work in Valle Imagna.

Species	Use Described in the Manuscript [17]	Use Described in Valle Imagna	Activity
**Amaryllidaceae**			
*Allium sativum* L. Garlic	Wine decoction with garlic, drunk against hip pain. Eaten raw against gout.	Ointment made of smashed garlic and pork fat to be externally applied against contusions and pain.	Anti-inflammatory for the treatment of musculoskeletal problems.
**Asteraceae**			
*Artemisia absinthium* L. Absinth	Pills or aqueous preparation with absinth salts (obtained from the ashes of *A. absinthium*) as diuretic and antipyretic. Powdered absinth mixed with honey. Kept inside the mouth as an anti-inflammatory for the tongue.	Infusion of the leaves or the entire above ground part drunk as an antipyretic, anti-inflammatory, and diuretic.	Anti-inflammatory, antipyretic, and diuretic.
*Matricaria chamomilla* L. Chamomile	Chamomile oil mixed with other ingredients, clysters against hip pain and sciatica.	Macerated chamomile oil, applied externally against muscular pain and inflammation.	Anti-inflammatory for the treatment of musculoskeletal problems.
**Brassicaceae**			
*Brassica oleracea* L. Cabbage	Cabbage leaves dried in the oven, powdered, and mixed with pork fat. The ointment is applied against hip pain.	Fresh leaves, smashed and applied on contusions and joint pain and inflammations.	Anti-inflammatory for the treatment of musculoskeletal problems.
**Lamiaceae**			
*Salvia officinalis* L. Sage	Sage leaves, rosemary, and pomegranate boiled in wine. Mouthwashes against painful and loosening teeth.	Sage leaves rubbed on teeth and gums as an anti-inflammatory. Sage infusion used as an anti-inflammatory and disinfectant mouthwash for teeth and gums.	Antibacterial Anti-inflammatory
**Linaceae**			
*Linum usitatissimum* L. Flax	Flax and fenugreek seeds boiled in water. The seeds are squeezed, and the mucilaginous water is mixed with butter. The ointment is applied on the chest against children cough.	Flax seeds boiled in water until a preparation similar to porridge is obtained. The mucilaginous poultice is applied warm on the chest against cough.	Antitussive Expectorant
**Oleaceae**			
*Olea europaea* L. Olive	An ointment of olive oil and bee wax or of olive oil and tallow applied on fissured hands and feet and on burns.	An ointment of olive oil and bee wax applied on burns and as soothing agent on inflamed skin.	Anti-inflammatory Soothing Wound healing
**Papaveraceae**			
*Chelidonium majus* L. Greater celandine	Latex of celandine with latex of parsnip applied on warts.	Latex of celandine applied on warts.	Antiviral Caustic
**Plantaginaceae**			
*Plantago* spp. *Plantago major* L. *Plantago lanceolata* L. Plantain	Poultice of plantain mixed with butter (and other ingredients) for two different ointments applied on inflamed nipples and on wounds.	Leaves applied externally on burns and as a wound healing and anti-inflammatory agent.	Anti-inflammatory Disinfectant Wound healing
**Rosaceae**			
*Agrimonia eupatoria* L. Agrimony	Complex remedy applied on deep leg ulcers. Above ground parts of agrimony and dried roses are boiled in wine, which is then used to disinfect the ulcers. A mix of powdered herbal and mineral ingredients are then applied on the wounds. Finally, the sediment of cooked agrimony and rose is smeared over the powder, and all is set in place with a gauze.	Fresh leaves are applied externally on deep wounds and set in place with a gauze. Compresses of the infusion of leaves or of above ground parts of agrimony are applied on deep wounds.	Anti-inflammatory Disinfectant Wound healing
*Rosa canina* L. Dog rose	Rose water, obtained from the petals, mixed with other ingredients and drunk in order to ‘refresh the kidneys’.	Infusions of the false fruits drunk as diuretic.	Diuretic
**Vitaceae**			
*Vitis vinifera* L. *Vitis* spp. Grapevine	Mouth washes with wine in which sage, rosemary, and pomegranate were boiled.	Mouth washes with grappa.	Anti-inflammatory Disinfectant (Alcohol?)

## Data Availability

This paper contains all data concerning the medicinal uses detected during the field work, as well as the one concerning the comparison among sources.

## Supplementary Materials

The following supporting information can be downloaded at: https://www.mdpi.com/article/10.3390/plants12142748/s1, Table S1: Traditional medicinal uses in 21st century Valle Imagna and related bibliographic research. For the complete reference list, please see the main text.
