# Supplementary material for: Remnants from the Past: From an 18th Century Manuscript to 21st Century Ethnobotany in Valle Imagna (Bergamo, Italy)
Source: Plants (Basel). 2023 Jul 24;12(14):2748. doi: 10.3390/plants12142748 (PMC10386062; doi:10.3390/plants12142748)
Supplement: Supplementary file 1 [file plants-12-02748-s001.zip › plants-2508465-supplementary.pdf]

Table S1: Traditional medicinal uses in 21st century Valle Imagna and related bibliographic research. For the complete reference list, please see the main text. CS = Circulatory system disorders; DIG = Digestive tract disorders; EAR = Afflictions of the ear; FE = Fever; GC = General condition; GYN = Gynaecological disorders, obstetric, and puerperal problems; MSK = Musculoskeletal system disorders and traumas; NS = Nervous system disorders; OR = Oropharyngeal cavity affections; OPT = Ophthalmic ailments; RES = Respiratory tract infections; UR = Urinary tract disorders; SK = Skin diseases and traumas; SL = Slimming.

**Scientific name, italian/english common name, vernacular name**	**Traditional uses in Valle Imagna**				**Bibliographic research: Ethnobotany**	**Bibliographic research: Biological activity**	**Bibliographic research: Phytochemistry linked to the biological activity**
	**Part of plant and preparation**	**Category of use/Apparatus**		**Detailed use**			
**Amaranthaceae**							
Blitum bonus-henricus (L.) Rchb. Buon Enrico/Wild spinach Parüc	Leaves (infusion)	Med	OR	Gingivitis, toothache, aphthae, and abscesses			
	Leaves (infusion)	Med	RES	Cough			
	Leaves (infusion)	Med	GC	Depurative			
**Amaryllidaceae**							
Allium cepa L. Cipolla/Onion Scigóla	Underground organs (externally as it is)	Med	SK	Insect bites	[33,44–46]	Anti-inflammatory, antibacterial [47]<br>Anti-allergic effect [48]	
Allium sativum L. Aglio/Garlic Aj	Underground organs (ointment)	Med	MSK	Contusions		Anti-inflammatory (oral use) [49,50]	Anti-inflammatory: Alliin, diallyl sulfide, diallyl disulfide, diallyl trisulfide, ajoene e S-allyl-cysteine [49,50]
Allium ursinum L. Aglio ursino/Ursin garlic	Leaves (preparation similar to pesto sauce)	Med	GC	Tonic and corroborant			
**Apiaceae**							
Angelica sylvestris L.	Fruits, infructescence	Med	GC	Anti-inflammatory		Anti-inflammatory [51]	

Angelica/Wild angelica	s, accessory fuits (infusion)						
Daucus carota L. Carota/Carrot	Underground organs (decoction)	Med	GC	Anti-inflammatory			
	Underground organs (decoction)	Med	GC	Depurative			
Petroselinum crispum (Mill.) Fuss Prezzemolo/Parsley	Leaves (applied as they are)	Med	SK	Insect bites	[44,45]		
**Araliaceae**							
Hedera helix L. Edera/Poison ivy	Leaves (macerated oil)	Med	CS	Improves circulation	[33] (Different preparation)	[52]	Hederagenin, oleanolic acid. [52]
**Asparagaceae**							
Asparagus officinalis L. Asparago/Asparagus Spàres	Shoots (eaten cooked)	Med	DIG	Liver depurative		[53]	
Polygonatum multiflorum (L.) All. Sigillo di Salomone/ David's harp	Leaves (applied as they are)	Med	SK	Anti-inflammatory, disinfectant, wound healing			
	Underground organs (hydroalcoholic macerated, applied externally)	Med	MSK	Contusions			
**Aspleniaceae**							

Ceterach officinarum Willd. Spaccapietra/ Rustyback	Underground organs (hydroalcoholic macerated, applied externally)	Med	MSK	Contusions			
				**Asteraceae**			
Achillea millefolium L. Achillea millefoglio/Common yarrow	Flowers, inflorescences, flowering tops (infusion)	Med	CS	Blood depurative	[34]		Anti-inflammatory: Sesquiterpene santamarin: [54]<br>Anti-inflammatory: Dicaffeoylquinic acids, flavonoids, proazulenic sesquiterpene lactone (matricin) [55]<br>Anti-bacterial: E.O. [55]<br>Potential estrogenic activitiy: flavonoids (apigenina, luteolin) [55]<br>Anti-bacterial, antioxidant and anti-nociceptive: E.O. (terpinolene, 1,8-cineol, thujone, γ-terpinene, borneol, camphor), rutin
	Flowers, inflorescences, flowering tops (infusion)	Med	GYN	Menstrual pain	[34,37]		
	Flowers, inflorescences, flowering tops (macerated oil)	Med	GYN	Menstrual pain			
	Flowers, inflorescences, flowering tops (infusion)	Med	GYN	Emmenagogue, regulates menstrual cycle		Emmenagogo, attività estrogenica [55–57]	
	Flowers, inflorescences, flowering tops (infusion)	Med	GC	Anti-inflammatory	[33]		

	Flowers, inflorescences, flowering tops (macerated oil)	Med	SK	Anti-inflammatory, disinfectant, wound healing	[33,34,37,58,59]	Antinfiammatoria, antibatterica [55] Antibatterica [56]	Anti-inflammatory: apigenin 7-O-glucoside, luteolin 7-O-glucoside<br><br>Antispasmodic: quercetin, apigenin e luteolin<br><br>Choleretic: luteolin-7-O-beta-D-glucuronide<br><br>Estrogenic activity: dihydrodehydrodiconiferyl alcohol, and 9-O-beta-D-glucopyranoside [56]
	Flowers, inflorescences, flowering tops (macerated oil)	Med	SK	Anti-inflammatory, emollient, soothing, redness	[33,34,37,58,59]	Antinfiammatoria, antibatterica [55]	
	Flowers, inflorescences, flowering tops (macerated oil)	Med	MSK	Contusions Ematomi	[34]	Antinfiammatoria [54–56]	
	Flowers, inflorescences, flowering tops (macerated oil)	Med	MSK	Pain and inflammation	[33,34]	Antinfiammatoria [54–56]	
	Above ground parts (infusion)	Med	UR	Diuretic	[37]		
Arctium lappa L. Bardana/ Greater burdock	Leaves (externally appliedente)	Med	MSK	Joint pain and inflammation	[36,37]		
Arnica montana L. subsp. *montana*	Flowers, inflorescences, flowering tops	Med	MSK	Contusions	[32–34,36,37,59,60]	Anti-inflammatory [61–64]	Analgesic and anti-inflammatory: sesquiterpene lactone helenanin [61,64–66]

Arnica montana/Mountain tobacco	(macerated oil alcoholic extract)						Anti-inflammatory: sesquiterpene lactones helenanin and dihydrohelenanin [61,62,67]
	Flowers, inflorescences, flowering tops (alcoholic extract or ointment)	Med	MSK	Pain and inflammation	[32–34,36,37,60]	Anti-inflammatory [61–64]	
	Flowers, inflorescences, flowering tops (compresses with infusion)	Med	MSK	Joint pain and inflammation	[32–34,36,37,60]	Anti-inflammatory [61–64]	
Artemisia absinthium L. Assenzio/Absinth Mistrù	Flowers, inflorescences, flowering tops (infusion)	Med	Other	Scurvy			Digestive: sesquiterpene lactones (absinthin, anabsinthin, arthabsin, anabsin) [67,68,70,71]
	Flowers, inflorescences, flowering tops Above ground parts (infusion)	Med	DIG	Digestive	[32–34,37,46,58–60]	[67–71]	
	Flowers, inflorescences, flowering tops (infusion)	Med	DIG	Carminative			
	Flowers, inflorescences	Med	UR	Kidney stones			

	, flowering tops (infusion)						
	Flowers, inflorescences, flowering tops (infusion)	Med	FE	Antipyretic	[33,46,60,72]	[73]	
	Leaves (infusion)	Med	NS	Sedative hypnotic, promotes sleep		[74]	
	Above ground parts (decoction)	Med	INF	Laxative in inflants			
	Above ground parts (infusion)	Med	UR	Diuretic			
	Above ground parts (infusion)	Med	GC	Anti-inflammatory		Anti-inflammatory and antioxidant [75]	
Calendula officinalis L. Calendula/Marigold	Flowers, inflorescences, flowering tops (alcoholic extract, macerated oil, applied as they are)	Med	SK	Anti-inflammatory, emollient, soothing, redness	[33,34,37,44,46]	[67,76–79]	Anti-inflammatory: quercetin [78]<br><br>Anti-inflammatory: triterpenoid esthers (faradiol-3-O-laurate, faradiol-3-O-palmitate efaradiol-3-O-myristate) Inhibition of collagen degradation: quercetin Stimulation of primary fibroblasts: quercetin-3-O-glucoside and rutin [77]
	Flowers, inflorescences, flowering tops (macerated oil)	Med	SK	Sunburn, burns, frostbites, redness, and rash	[33,34,37,44]	[67,76–79]	

	Flowers, inflorescences, flowering tops (alcoholic extract)	Med	MSK	Contusions		-	Wound healing: carotenoids [80] Anti-inflammatory and anti-edema: terpenoids and flavonoids [81]
	Flowers, inflorescences, flowering tops (macerated oil)	Med	MSK	Muscle pain and inflammation	[34]	-	
	Flowers, inflorescences, flowering tops (infusion drunk, macerated oil applied)	Med	NS	Sedative hypnotic, promotes sleep			
	Flowers, inflorescences, flowering tops (infusion)	Med	GC	Anti-inflammatory			
Centaurea benedicta (L.) L. Cardo benedetto/ Blessed thistle	Flowers, inflorescences, flowering tops (decoction)	Med	CS	Improves cardiac functions			
	Flowers, inflorescences, flowering tops (decoction)	Med	DIG	Digestive			

Cyanus segetum Hill Fiordaliso/ Bachelor's button	Flowers, inflorescences, flowering tops (infusion, compresses)	Med	OPT	Eye anti-inflammatory	[36,60]		
Helianthus tuberosus L. Topinambur/ Jerusalem artichoke	Underground organs (eaten cooked)	Med	DIG	Liver depurative		[82]	
	Underground organs (eaten cooked)	Med	UR	Diuretic			
Matricaria chamomilla L. Camomilla/Chamomile	Flowers/inflorescences/flowering tops (infusion)	Med	DIG	Digestive	[33,44,45]	Attività antispasmodica [76,83]	Anti-inflammatoy: apigenin [67,76,83,84] Anxiolytic and antispasmodic: apigenin [67,83,85] Antispasmodic: bisabolol [83] Anti-inflammatory: bisabolol [76] Anti-inflammatory and spasmolytic: matricin [76]
	Flowers/inflorescences/flowering tops (infusion)	Med	NS	Sedative hypnotic, promotes sleep	[32–34,36,37,44–46]	Attività ansiolitica [67,83]	
	Flowers/inflorescences/flowering tops (macerated oil)	Med	SK	Anti-inflammatory, emollient, soothing, redness	[33]	Anti-inflammatory, wound healing [86]	
	Flowers/inflorescences/flowering tops (macerated oil)	Med	SK	Sunburn, burns, frostbites, redness, and rash	[33]	Anti-inflammatory, wound healing [86]	
	Flowers/inflorescences/flowering tops	Med	MSK	Muscle pain and inflammation	[33]	[86]	

	(macerated oil)						
Silybum marianum (L.) Gaertn. Cardo mariano/Milk thistle	Flowers/inflorescences/flowering tops (decoction)	Med	CS	Improves cardiac functions		[87]	Cardioprotective: silymarin [87]
Taraxacum sect, *Taraxacum* Tarassaco/Dandelion	Flowers/inflorescences/flowering tops (decoction)	Med	DIG	Digestive			Digestive: bitter sesquiterpene lactones [88]<br><br>Cholesterol: triterpenes and phytosterols (β-sitosterol and amidiol) [89]<br><br>Prebiotics: polysaccharides [88]<br>Hepatoprotective: complex polysaccharides (inulin) [88]
	Flowers/inflorescences/flowering tops (infusion)	Med	DIG	Digestive	[36,45]	[88]	
	Flowers/inflorescences/flowering tops (infusion)	Med	DIG	Liver depurative	[36,37]		
	Flowers/inflorescences/flowering tops (syrup)	Med	RES	Sore throat and hoarseness<br>Cough	[33,37]		
	Flowers/inflorescences/flowering tops (syrup)	Med	GC	Depurative	[33]		
	Leaves (decoction, eaten cooked)	Med	CS	Blood depurative	[36]	Decreases cholesterol absorption [89]	
	Leaves (decoction, infusion, eaten cooked)	Med	DIG	Liver depurative	[36,46]	Hepatoprotective [88]	

	Leaves (eaten cooked)	Med	DIG	Laxative, bowel transit	[90]	Prebiotics [88]	
	Leaves (eaten cooked)	Med	UR	Diuretic	[36]	Diuretic [89]	
	Underground organs (eaten cooked)	Med	CS	Blood depurative	[32,36,60]	Decreases cholesterol absorption [89]	
	Underground organs (decoction)	Med	DIG	Liver depurative	[32,36,44]	Liver disorders [89] Hepatoprotective [88]	
	Underground organs (decoction)	Med	GC	Tonic and corroborant			
Tussilago farfara L. Tossilaggine/ Coltsfoot	Flowers/inflorescences/flowering tops (infusion)	Med	RES	Cough	[33,36,37,59,60]	Antitussivo, espettorante [91,92]	Antitussive: Caffeoylquinic acids from flowers [92]
	Leaves Underground organs (alcoholic macerate, externally applied)	Med	MSK	Contusions			Antitussive: Terpenoids, organic acids, flavonoids, alkaloids, chromones, volatile oil in the flowers [91]
				**Betulaceae**			
Betula pendula Roth Betulla/Birch	Fusti legnosi/rami/rametti/polloni (used as whip)	Med	CS	Improves circulation			
	Latice o linfa (drunk as it is)	Med	UR	Diuretic Depurative			

	Latice o linfa (drunk as it is)	Med	GC	Tonic and corroborant	[34]	[93]	
**Boraginaceae**							
Symphytum officinale L. Consolida maggiore/Comfrey	Leaves (poultice with green clay)	Med	NS	Sciatica and neuralgia			
	Leaves (compresses with infusion)	Med	MSK	Fractures			
	Leaves (compresses with infusion)	Med	MSK	Joint pain and inflammation	[60]	[94,95]	
**Brassicaceae**							
Brassica oleracea L. Verza/Savoy cabbage Vérz	Leaves (Externally applied)	Med	MSK	Arthrosis<br>Rheumatic pains<br>Joint pain and inflammation	[34,36,44,45]	Anti-inflammatory (several different *var.*) [96]	Anti-inflammatory: nicotin [96]
	Leaves (Externally applied)	Med	MSK	Contusions	[34]		
Brassica rapa L. Rapa/Turnip	Underground organs (eaten cooked)	Med	GC	Depurative			
Diplotaxis tenuifolia (L.) DC. Rucola selvatica/Wild rocket	Leaves (eaten raw)	Med	DIG	Digestive	[44]		
	Leaves (eaten raw)	Med	DIG	Laxative, bowel transit			
**Convolvulaceae**							
Convolvulus arvensis L.	Above ground parts (infusion)	Med	MSK	Contusions			

Convolvolo/Bindweed							
**Cornaceae**							
Cornus mas L. Corniolo/Dogwood Cornàl	Fruits/infructescences/accessory fruits (eaten as they are)	Med	GC	Tonic and corroborant		Vitamin C content: 34–100 mg/100 g fresh weight [97]	
**Cupressaceae**							
Juniperus communis L. Ginepro/Juniper Züerna	Galbule (chewed as they are)	Med	DIG	Stomach anti-inflammatory	[45]		
	Galbule (alcoholic macerate)	Med	OR	Gingivitis, toothache, aphthae, and abscesses	[46]		
**Equisetaceae**							
Equisetum arvense L. Equiseto/Horsetail	Above ground parts (infusion)	Med	GC	Depurative			
	Above ground parts (as aromatic salt)	Med	GC	Tonic and corroborant	[33,34,36,37]	Remineralizing [98]	High concentrations minerals: [98]
	Above ground parts (infusion)	Med	CS	Hypotensive		Diuretic [98–100]	
**Ericaceae**							
Rhododendron ferrugineum L. Rododendro/ Rhododendron	Flowers/inflorescences/flowering tops (honey)	Med	RES	Sore throat and hoarseness			
Vaccinium myrtillus L. Mirtillo nero/Blueberry	Fruits/infructescences/accessory fruits	Med	OPT	Improves sight and eye health	[33,36,37,46]	WHO Monograph - Fructus Myrtilli) [101] [102–107] (Refuted by [108])	Improve ocular microcirculation: phenolic compounds (specifically anthocyanins) [101,109]

	(eaten as they are)						Protection from retinal damage: cyanidin, delphinidin, and malvidin [105]
			**Euphorbiaceae**				
Ricinus communis L. Ricino/Castor	Seeds (castor oil, drunk)	Med	SK	Anti-inflammatory, emollient, soothing, redness			Laxative: ricinoleic acid [110]
	Seeds (castor oil, drunk)	Med	DIG	Laxative, bowel transit		[110]	
			**Fabaceae**				
Phaseolus vulgaris L. Fagiolo/Bean	Legume exocarp (decoction)	Med	MET	Hypocholesterolemic		(Black seed variety)[111]	Flavonoids and saponins (black seeds) [111]
			**Gentianaceae**				
Gentiana acaulis L. Gentianella/Trumpet gentian	Underground organs (alcoholic macerate, rub on temples)	Med	NS	Headache			
			**Hypericaceae**				
Hypericum perforatum L. Iperico/St. John's wort	Flowers/inflorescences/flowering tops (macerated oil, secreted directly applied)	Med	SK	Anti-inflammatory, disinfectant, wound healing<br><br>Anti-inflammatory, emollient, soothing, redness<br><br>Insect bites<br><br>Sunburn, burns, frostbites, redness, and rash	[32–37,44–46,58,60,112]	WHO Monograph – Herba Hyperici [54] [67,112]	Antimicrobial: hyperforin, hypericin, and pseudohypericin [112]<br><br>Antioxidant and anti-inflammatory: hyperforin and flavonoids [112]

	Flowers/inflorescences/flowering tops (macerated oil)	Med	MSK	Contusions	[32–34,36,37,46,58,60,112]	[112,113]	
	Flowers/inflorescences/flowering tops (macerated oil)	Med	MSK	Arthritis<br>Rheumatic pains	[32–37,46,58,60,112]	[112,113]	
	Flowers/inflorescences/flowering tops (macerated oil)	Med	RES	Facilitates respiration			
				**Juglandaceae**			
Juglans regia L.<br>Noce/Walnut	Leaves (infusion for baths)	Med	INF	Anti-inflammatory, emollient, soothing, redness		[114]	
	Seeds (oil)	Med	EAR	Otitis and pains			
				**Lamiaceae**			
Lavandula angustifolia Mill.<br>Lavanda/Lavender	Flowers/inflorescences/flowering tops (vinegar macerate)	Med	SK	Hands and feet mycosis		[115]	Wound healing: linalool and linalyl acetate [116]<br>Psoriasis: essential oil, specifically linalool and linalyl acetate as main compounds [117]<br>Sedative: essential oil [115,118–120]
	Flowers/inflorescences/flowering tops (macerated oil)	Med	SK	Anti-inflammatory, emollient, soothing, redness<br><br>Sunburn, burns, frostbites, redness, and rash		Wound healing [115,116]	

Flowers/inflorescences/flowering tops (macerated oil)	Med	SK	Psoriasis		[115,117]<br>[121] (a case of psoriasis allegedly replaced by allergic dermatitis)
Flowers/inflorescences/flowering tops (macerated oil)	Med	MSK	Muscle pain and inflammation	[46]	Anti-inflammatory [122]
Flowers/inflorescences/flowering tops (macerated oil)	Med	NS	Mal di testa	[46]	[123]<br>[124] (headache as one of the adverse reactions to lavender essential oil)
Flowers/inflorescences/flowering tops (macerated oil, infusion)	Med	NS	Sedative hypnotic, promotes sleep	[33,36,44]	[115,118–120]
Flowers/inflorescences/flowering tops (infusion)	Med	DIG	Antispasmodic (stomach)		Antispasmodic, ileum [125]
Flowers/inflorescences/flowering tops (infusion)	Med	DIG	Digestive		
Flowers/inflorescences/flowering tops (infusion) (with thyme, lemon and ginger)	Med	RES	Facilitates respiration	[33,36,46]	[126,127]

Melissa officinalis L. Melissa/Lemonbalm	Flowers/inflorescences/flowering tops Leaves (infusion)	Med	NS	Sedative hypnotic, promotes sleep	[33,35–37,44]	[128]	Digestive: rosmarinic acid [129,130]
	Leaves (infusion)	Med	NS	Antidepressant		[128]	
	Leaves (infusion)	Med	DIG	Digestive	[33,35,45,46]	[129,130]	
Mentha spp Menta/Mint	Leaves (applied as they are)	Med	SK	Insect bites	[44]	Antinociceptive [131]	Anti-inflammatory: flavonoids [131] Sedative: mainly phenolic compounds (flavonoids and tannins) [132] Respiratory problems: essential oil [133]; cis-piperitone epoxide, piperitenone oxide, pulegone, and menthone [131] Oropharyngeal cavity: flavonoids; menthol [131]
	Leaves (decoction)	Med	GC	Anti-inflammatory	[34,37]	[131,134–137]	
	Leaves (infusion)	Med	NS	Sedative hypnotic, promotes sleep	[33,36]	[132,136]	
	Leaves (infusion, alcoholic macerate)	Med	RES	Cough	[32,33]	Antibacterial: [138] [133] (mix of essential oils, *M. piperita*)	
	Leaves (inhalation of the infusion)	Med	RES	Facilitates respiration	[33]	[133] (mix of essential oils, *M. piperita*)	
	Leaves (alcoholic macerate)	Med	OR	Gingivitis, toothache, aphthae, and abscesses	[33]	Antinociceptive; anti-inflammatory, antibacterial against *Staphylococcus mutans* [131] Anti-inflammatory [135–137,139]	
Plectranthus amboinicus (Lour.) Spreng.	Leaves (decoction)	Med	RES	Asthmatic bronchitis Cough	[140,141] (where indigenous)	[140]	

Origano Cubano/Cuban oregano							
Rosmarinus officinalis L. Rosmarino/Rosemary	Flowers/inflorescences/flowering tops (infusion, drunk or for baths)	Med	GC	Tonic and corroborant	[33,36,44]	Potentially immunomodulating activities [142] Antidepressant-like properties [143]	Anti-inflammatory, antinociceptive, antifungal, wound healing: carnosol; carnosic, rosmarinic, ursolic, oleanolic, and micromeric acids. E.O.: β- pinene, 1,8-cineole, borneol, camphor, limonene, and verbenone [144] Respiratory conditions: carnosic acid [145]
	Leaves (vinegar macerate)	Med	SK	Hands and feet mycosis		Antifungal [144]	
	Leaves (inhalations with infusion)	Med	RES	Expectorant, decongestant, emollient	[36,45,46]	[145] [133] (mix of essential oils, *R. officinalis*)	
	Leaves (infusion)	Med	DIG	Stomach anti-inflammatory	[146,147]	Anti-inflammatory [144]	
	Leaves (infusion)	Med	UR	Diuretic	[148]	[149]	
	Leaves (infusion)	Med	GC	Anti-inflammatory		Anti-inflammatory [144]	
	Leaves (infusion, alcoholic macerate)	Med	GC	Tonic and corroborant	[33,36,44]	Potentially immunomodulating activities [142] Antidepressant-like properties [143]	
	Leaves (ointment)	Med	SK	Anti-inflammatory, disinfectant, wound healing Sunburn, burns, frostbites, redness, and rash	[44]	[144]	

	Flowers/inflorescences/flowering tops (infusion, drunk or for baths)	Med	GC	Tonic and corroborant	[33]		
	Leaves (applied as they are, infusion)	Med	OR	Disinfettante Gingivitis, toothache, aphthae, and abscesses	[33,36,46,60]	[151,153,155]	Anti-inflammatory action: flavonoids, rosmarinic acid, manool, carnosol, ursolic acid [150] Antimicrobial for oral cavity affections, anti-inflammatory: 1,8-cineole, thujone, and camphor [151,152] Antifungal for oral cavity affections: E.O. [153]
	Leaves (vinegar macerate)	Med	SK	Micosi mani e piedi			
Salvia officinalis L. Salvia/Sage	Leaves (applied as they are)	Med	SK	Insect bites	[36]	Anti-inflammatory [151,154] Wound healing [156]	
	Leaves (decoction)	Med	GC	Depurative			
	Leaves (decoction, infusion)	Med	GC	Anti-inflammatory		Anti-inflammatory [151,154]	Anti-inflammatory: ursolic acid and other diterpenes [151]
	Leaves (infusion)	Med	DIG	Stomach anti-inflammatory	[46]	Anti-inflammatory [151,154]	Anti-inflammatory: MeOH extract rich in rosmarinic, ursolic, caffeic, and oleanolic acids; borneol [154]
	Leaves (infusion, drunk or for baths, alcoholic macerate)	Med	GC	Tonic and corroborant	[33]		
	Leaves (ointment)	Med	SK	Anti-inflammatory, disinfectant, wound healing		Anti-inflammatory [151,154] Wound healing [156]	

				Sunburn, burns, frostbites, redness, and rash			
Thymus spp. Timo/Thyme	Leaves (inhalations with infusion)	Med	RES	Expectorant, decongestant, emollient	[33,35,36,46,90] [37,44] (External use)	[157–160]	Antibacterial, antioxidant: phenolic compounds, carvacrol, thymol [158,161] Antibacterial: E.O. (specifically thymol) [159] Anti-inflammatory: thymol [162]
	Leaves (infusion)	Med	DIG	Antispasmodic (stomach)	[59,158,159,163–166]	Spasmolytic (ileum and colon) [167]	
	Leaves (infusion) Alone or with lemon, ginger, and lavender	Med	DIG	Digestive	[33,35,58,60]		
	Leaves (compresses with infusion)	Med	MSK	Contusions	[163]	Anti-inflammatory [162]	
	Leaves (infusion) Alone or with lemon, ginger, and lavender	Med	RES	Bronchitis Expectorant, decongestant, emollient Sore throat and hoarseness Facilitates respiration	[32,33,35–37,44,46,59,60,158,159,163,164,166,168]	[157–160]	
	Leaves (infusion)	Med	GC	Anti-inflammatory	[158]	Anti-inflammatory [162]	
	Leaves (ointment)	Med	SK	Anti-inflammatory, disinfectant, wound healing Sunburn, burns, frostbites, redness, and rash	[33]	Antibacterial and wound healing [169] Anti-inflammatory [159,162] Antioxidant [158]	
**Lauraceae**							
Laurus nobilis L.	Leaves	Med	RES	Expectorant, decongestant, emollient	[36]		Anti-inflammatory: EO [170]; 1,8-cineole [171]

Alloro	(inhalations with infusion)					
	Leaves (decoction)	Med	GC	Anti-inflammatory		Anti-inflammatory [170,171]
	Leaves (decoction)	Med	GC	Depurative		
	Leaves (infusion)	Med	OTH	Aftermath of drunkennes		
	Leaves (infusion)	Med	CS	Ipotensivo<br>Migliora la funzionalità cardiaca		Antihypertensive [172]
	Leaves (infusion, drunk or for baths, alcoholic macerate, external)	Med	MSK	Rheumatic pains<br>Pain and inflammation<br>Muscle pain and inflammation		Anti-inflammatory [171]
	Leaves (infusion)	Med	NS	Mal di testa	[32] (External use)	
**Linaceae**						
Linum usitatissimum L.<br>Lino/Flax	Seeds (flax flour mixed with hot water, appliled in the chest)	Med	RES	Expectorant, decongestant, emollient<br>Cough	[35–37,46,59,60] [90] (Oral use) [34](Above ground parts)	
**Malvaceae**						
Alcea rosea L.<br>Malvone/Hollyhocks	Above ground parts (infusion)	Med	RES	Sore throat and hoarseness		

Malva sylvestris L. Malva/Mallow	Flowers/inflorescences/flowering tops Leaves Above ground parts (infusion, decoction, drunk or compresses and mouthwashes)	Med	OR	Anti-inflammatory Gingivitis, toothache, aphthae, and abscesses Tonsillitis	[34–36,45,46]	[173–176]	Anti-inflammatory: flavonoids and mucilages [175] Anti-inflammatory: mucilaginous complex [177] Anti-inflammatory: polyphenols [173] Anti-inflammatory: Malvidin-3-glucoside [178]
	Flowers/inflorescences/flowering tops Leaves Above ground parts (infusion)	Med	DIG	Bowel anti-inflammatory Bowel depurative Belly ache Laxative, bowel transit	[33,34,36,45,46,90]	Anti-inflammatory [176]	
	Leaves (infusion)	Med	DIG	Digestive Stomachache Antispasmodic (stomach)	[34,60]		
	Leaves (infusion)	Med	NS	Sedative hypnotic, promotes sleep	[34]	[175]	
	Leaves (eaten cooked, in soup)	Med	GC	Depurative			
	Flowers/inflorescences/flowering tops Leaves Above ground parts	Med	GC	Anti-inflammatory		Anti-inflammatory [176]	

(infusion, decoction)					
Leaves (externally apllied on the cheek)	Med	OR	Gingivitis, toothache, aphthae, and abscesses	[45]	
Leaves (decoction)	Med	SK	Hands and feet mycosis		
Leaves (infusion)	Med	SK	Anti-inflammatory, disinfectant, wound healing<br>Anti-inflammatory, emollient, soothing, redness<br>Sunburn, burns, frostbites, redness, and rash	[33–36,46]	[175,176,178–181]
Leaves Above ground parts (infusion)	Med	SL	Slimming		
Leaves (infusion, drunk or vaginal douche)	Med	UR	Cystitis and other urinary tract inflammations<br>Depurative<br>Diuretic	[60]	[176,182]
Leaves (compresses with infusion)	Med	MSK	Joint pain and inflammation	[34]	Anti-inflammatory [176]
Leaves (infusion, vaginal douche)	Med	GYN	Anti-inflammatory and soothing, redness	[33–36]	Anti-inflammatory [176]
Leaves (infusion)	Med	RES	Bronchitis	[33,34,36]	[183] (Herbal mixture)

				Expectorant, decongestant, emollient<br>Cold<br>Cough	
	Leaves (infusion)	Med	GC	Infections	
	Above ground parts (infusion)	Med	CS	Hypotensive	
Tilia cordata Mill<br>Or<br>*Tilia platyphyllos* Scop.<br>Tiglio/Limetree<br>Fiur de tej	Flowers/inflorescences/flowering tops<br>Leaves (decoction, infusion, bevuti o suffumigi, syrup)	Med	RES	Respiratory tract anti-inflammatory<br>Expectorant, decongestant, emollient<br>Sore throat and hoarseness<br>Facilitates respiration<br>Cold<br>Cough	[33–35,37,46,58,60]
	Flowers/inflorescences/flowering tops (compresses con infusion)	Med	SK	Anti-inflammatory, emollient, soothing, redness<br>Sunburn, burns, frostbites, redness, and rash	
	Flowers/inflorescences/flowering tops (infusion)	Med	DIG	Antiacid, gastritis, and reflux<br>Ulcer	
	Flowers/inflorescences/flowering tops (infusion)	Med	DIG	Digestive<br>Stomachache	[33,34]
	Flowers/inflorescences/flowering tops	Med	NS	Sedative hypnotic, promotes sleep	[32,33,35]

	Leaves (infusion)						
	Flowers/inflorescences/flowering tops<br>Leaves (infusion)	Med	GC	Anti-inflammatory			
				**Moraceae**			
Ficus carica L.<br>Fico/Fig	Latice (applied)	Med	SK	Warts and tags	[45,46]	[184]	Anti-warts: proteolytic enzymes [184]
				**Myrtaceae**			
Eucalyptus globulus Labill.<br>Eucalipto/Eucalyptus	Leaves (decoction, inhalations with the infusion)	Med	RES	Cold<br>Cough	[141]	Airways anti-inflammatory, mucolytic, spasmolytic [185]	Airways anti-inflammatory, mucolytic, spasmolytic: 1,8-cineole [185]
Syzygium aromaticum (L.) Merr. & L.M.Perry<br>Chiodi di garofano/Cloves	Flowers/inflorescences/flowering tops (applied on the tooth)	Med	OR	Gingivitis, toothache, aphthae, and abscesses	[186,187]	[188]	Antimicrobial: Eugenol [188]
				**Oleaceae**			
Olea europaea L.<br>Olivo/Olive tree	Leaves (infusion)	Med	GC	Anti-inflammatory		Anti-inflammatory (specifically, intestine) [189]<br><br>Anti-inflammatory [190]	Leaves:<br><br>Anti-inflammatory: Hydroxytyrosol [189]<br><br>Anti-inflammatory: Oleanolic acid [190]<br><br>Olive oil:
	Fruits/infructescences/accessory fruits (oil)	Med	EAR	Otitis and pain	[33,46]	[193]<br>(Not enough evidence)	

	Fruits/infructescences/accessory fruits (oil and bee wax based ointment)	Med	SK	Anti-inflammatory, disinfectant, wound healing<br>Sunburn, burns, frostbites, redness, and rash	[45,46]	Anti-inflammatory and wound healing [191,192]	Anti-inflammatory and wound healing: polyphenols [191,192]
**Papaveraceae**							
Chelidonium majus L.<br>Chelidonio/Celandine<br>Erba dei porri	Leaves and stemsi, latex (applied on the part)	Med	SK	Anti-inflammatory, disinfectant, wound healing<br>Verruche e porri	[33–37,44–46,60]	[194,195]	
**Pinaceae**							
Abies alba Mill.<br>Abete bianco/European silver fir	Resin (applied as it is)	Med	SK	Anti-inflammatory, disinfectant, wound healing	[60]		
	Resin (ointment)	Med	MSK	Contusions	[32,60]		
Picea abies (L.) H.Karst.<br>Abete rosso/European spruce<br>Pìgher	Resin (applied as it is)	Med	SK	Anti-inflammatory, disinfectant, wound healing	[34–36]	Antibacterial, wound healing [196,197]<br>Improves reepithelization [198]	Antibacterial, wound healing: diterpenes (abietic, neoabietic, dehydroabietic, pimaric, isopimaric, levopimaric, sandracopimaric, and palustric acids); lignans (pinoresinol, lariciresinol, and matairesinol); cinnamic acid [197]
Pinus mugo Turra	Immature cones, buds (syrup)	Med	RES	Sore throat and hoarseness<br>Cough	[32–37,60]	[199,200]	

Pino mugo/Dwarf mountain pine						
Pinus spp Pino/Pine	Resin (applied as it is)	Med	SK	Anti-inflammatory, disinfectant, wound healing	[33,46,60]	[198]	
**Plantaginaceae**						
Plantago lanceolata L. Piantaggine lanceolata/ Narrowleaf plantain	Leaves (applied on the part)	Med	SK	Insect bites	[32–37,58]	Anti-inflammatory [201]	Anti-inflammatory: maybe correlation with content of apigenin and ursolic acid [201]
	Leaves (applied on the part)	Med	SK	Sunburn, burns, frostbites, redness, and rash	[36,37]	[201–203]	
Plantago major L. Piantaggine maggiore/Great plantain	Leaves (applied on the part)	Med	SK	Insect bites	[44,58]	[201,204–206]	Anti-inflammatory: maybe correlation with content of apigenin and ursolic acid [201]
	Leaves (infusion)	Med	GC	Tonic and corroborant	[36]		
Plantago spp. Piantaggine/Plantain	Leaves (applied on the part)	Med	SK	Anti-inflammatory, disinfectant, wound healing	[32,33,36,37,45,46,58]	[201–206]	Anti-inflammatory: maybe correlation with content of apigenin and ursolic acid [201]
	Leaves (poultice applied on the stomach)	Med	DIG	Stomachache			
**Poaceae**						
Cynodon dactylon (L.) Pers. Gramigna/Spear grass Gramègna	Leaves (infusion)	Med	MSK	Arthritis		[207]	
	Underground organs (infusion)	Med	UR	Cystitis and other urinary tract inflammations	[33,45,46,60]	[208,209]	

Zea mays L.<br>Mais, granturco/Corn<br>Melgòt	Flowers/inflorescences/flowering tops (infusion)	Med	UR	Diuretic	[33,36]<br>Fruits [46]	[210]	
**Polygonaceae**							
Rumex acetosa L.<br>Acetosa/Common sorrel<br>Erba cœca	Leaves (eaten raw in the fields or in salads)	Med	DIG	Laxative, bowel transit	[37,90]		
Rumex alpinus L.<br>Romice alpino/Monk's-rhubarb	Leaves (directly applied)	Med	MSK	Pain and inflammation	[34, 37]		
Rumex obtusifolium L.<br>Romice comune/ Bitter dock	Leaves (directly applied)	Med	MSK	Contusions<br>Sprains			
**Polypodiaceae**							
Polypodium vulgare L.<br>Polipodio/ Common polypody	Underground organs (eaten raw)	Med	DIG	Liver depurative			
**Portulacaceae**							
Portulaca oleracea L.	Leaves	Med	GC	Tonic and corroborant		High content of vitamins, minerals [211]	Vitamins: vitamin A, ascorbic acid, α-tocopherol, niacin,

Portulaca, erba porcellana/Purslane	(eaten raw in the fields and in salads)						pyridoxine, and riboflavin. Minerals: phosphorus, manganese, iron, calcium, selenium. Amino acids: isoleucine, proline, leucine, lysine, phenylalanine, methionine, cystine, valine, threonine, and tyrosine [211]
				**Ranuncolaceae**			
Clematis vitalba L. Clematide/ Old man's beard Ligabosc	Germogli (eaten cooked)	Med	DIG	Laxative, bowel transit			
	Underground organs (externally applied)	Med	SK	Anti-inflammatory, disinfectant, wound healing			
Delphinium consolida L. Erba cornetta/ Larkspur	Leaves (poultice)	Med	MSK	Contusions			
	Leaves (Poultice)	Med	MSK	Fractures			
				**Rosaceae**			
Agrimonia eupatoria L. Agrimonia/Agrimony Erba del venìl	Leaves Above ground parts Whole plant (applied as it is, compresses with the infusion or the decoction)	Med	SK	Anti-inflammatory, disinfectant, wound healing	[33](dermatitis)	[212–216]	Anti-inflammatory (aerial parts): polyphenols (flavan-3-ols, quercetin, kaempferol, apigenin, and luteolin derivatives. Agrimoniin, p-coumaric acid, and ellagic acid) [217]

	Whole plant (compresses with the decoction)	Med	INF	Anti-inflammatory, emollient, soothing		Anti-inflammatory [215]
	Leaves (infusion)	Med	UR	Prostate gland		
	Leaves (compresses)	Med	MSK	Contusions		Anti-inflammatory [215]
	Leaves (infusion)	Med	GC	Anti-inflammatory		Anti-inflammatory [215]
	Above ground parts Whole plant (infusion, decoction)	Med	DIG	Stomachache	[46] (anti-inflammatory for the digestive tract) [44] (digestive)	
	Whole plant (decoction as mouthwashes)	Med	OR	Gingivitis, toothache, aphthae, and abscesses		Anti-inflammatory [215]
	Whole plant (decoction)	Med	DIG	Laxative, bowel transit		
	Whole plant (decoction, drunk or as vaginal douche)	Med	UR	Cystitis and other urinary tract inflammations		Anti-inflammatory [215]
	Whole plant (infusion)	Med	UR	Prostate gland		
Aronia melanocarpa (Michx.) Elliot Aronia/ black chokeberry	Fruits/infructescences/accessory fruits (eaten)	Med	DIG	Antinausea, antiemetic		

Crataegus monogyna Jacq.<br>Biancospino/Hawthorn	Flowers/inflorescences/flowering tops (infusion)	Med	CS	Improves cardiac function<br>Tachycardia, antiarrhythmic	[33,35,37,44,58]	Fruits [119]<br>[218]
	Flowers/inflorescences/flowering tops (infusion)	Med	CS	Hypotensive	[33,37,44]	[218]
	Flowers/inflorescences/flowering tops<br>Leaves (infusion)	Med	NS	Sedative hypnotic, promotes sleep	[33,37]	[218]
Malus sylvestris (L.) Mill.<br>Melo/Apple tree<br>Specific cultivar: Mela ruggine (Rusty apple)	Fruits/infructescences/accessory fruits (eaten raw or cooked)	Med	GC	Tonic and corroborant		
Prunus avium L.<br>Ciliegio/Cherry tree	Fruits' stalks (infusion, decoction)	Med	UR	Cystitis and other urinary tract inflammations<br>Diuretic	[35,46,60]	[219]
Prunus spinosa L.<br>Prugnolo selvatico/Blackthorn	Flowers/inflorescences/flowering tops (infusion)	Med	DIG	Laxative, bowel transit	[33] (fruits)	
	Flowers/inflorescences/flowering tops (infusion)	Med	UR	Diuretic		

	Flowers/inflorescences/flowering tops (infusion)	Med	RES	Expectorant, decongestant, emollient		
	Flowers/inflorescences/flowering tops (infusion)	Med	GC	Tonic and corroborant (rich in vitamin C)		Rich in vitamins, specifically vitamin C [220]
	Fruits/infructescences/accessory fruits (directly applied)	Med	SK	Anti-inflammatory, disinfectant, wound healing	[46]	[221,222]
	Branches rhytidome (decoction)	Med	FE	Antipyretic	[45]	
Rosa canina L.<br>Rosa canina/Dogrose<br>Grata Cül	Fruits/infructescences/accessory fruits (Eaten, infusions, jams)	Med	GC	Tonic and corroborant (Rich in vitamin C)	[33,90]	High Vitamin C contents [223]
	Fruits/infructescences/accessory fruits (infusion)	Med	CS	Varicose veins and related pain		
	Fruits/infructescences/accessory fruits (infusion)	Med	DIG	Stomachache		[223]
	Fruits/infructescences/accessory fruits (infusion)	Med	UR	Cystitis and other urinary tract inflammations Diuretic	[37]<br>[34] (leaves)	[224]

	Fruits/infructescences/accessory fruits (infusion)	Med	NS	Sedative hypnotic, promotes sleep	[34] (flowers)		
	Fruits/infructescences/accessory fruits (infusion)	Med	RES	Cough	[36]		
	Fruits/infructescences/accessory fruits (infusion)	Med	GC	Anti-inflammatory Antioxidant		Anti-inflammatory [223]	
	Fruits/infructescences/accessory fruits (infusion)	Med	GC	Immunomodulant (Traditionally attributed to Vitamin C contents)	[35,60]	High Vitamin C contents [223]	
Rosa spp Rosa	Flowers/inflorescences/flowering tops (eaten in salads)	Med	DIG	Liver depurative		[225] (False fruits *R. laevigata*)	Anti-inflammatory (flowers *R. rugosa*) Phenolic compounds [225]
	Flowers/inflorescences/flowering tops Macerated oil	Med	SK	Anti-inflammatory, emollient, soothing, redness Sunburn, burns, frostbites, redness, and rash	[45]	Anti-inflammatory (*R. rugosa*) [225]	
	Flowers/inflorescences/flowering tops Macerated oil	Med	MSK	Muscle pain and inflammation		Anti-inflammatory (*R. rugosa*) [225]	
Rubus ulmifolius Schott	Seeds (put in a satchel and heated)	Med	MSK	Muscle pain and inflammation	Similar use with cherry seeds in [34]	Heat therapy [226]	

Rovo da mora/Bramble				
Sorbus aria (L.) Crantz<br>Sorbo montano/ Common whitebeam	Flowers/inflorescences/flowering tops (honey)	Med	RES	Sore throat and hoarseness
Sorbus aucuparia L.<br>Sorbo degli uccellatori/ Mountain ash	Flowers/inflorescences/flowering tops (honey)	Med	RES	Sore throat and hoarseness
**Rutaceae**				
Citrus x *limon* (L.) Osbek<br>Limone/Lemon tree	Flowers/inflorescences/flowering tops (infusion)	Med	DIG	Antispasmodic (stomach)
	Fruits/infructescences/accessory fruits (infusion) (dandelion flowers in infusion with lemon, orange, and raisin)	Med	DIG	Digestive
	Fruits/infructescences/accessory fruits	Med	MSK	Sprains

(applied as it is)				
Fruits/infructescences/accessory fruits, juice (drunk as it is with castor oil)	Med	SK	Anti-inflammatory, emollient, soothing, redness	[44]
Fruits/infructescences/accessory fruits, peel Fruits/infructescences/accessory fruits, juice (eaten as it is or as a marmalade or as juice with castor oil)	Med	DIG	Laxative, bowel transit	
Fruits/infructescences/accessory fruits, juice (drunk as it is with honey and ginger)	Med	RES	Sore throat and hoarseness Facilitates respiration	[46]
Fruits/infructescences/accessory fruits, juice (drunk with thyme,	Med	DIG	Digestive Antispasmodic (stomach)	[46]

	ginger, and lavender)						
Citrus sinensis (L.) Osbeck<br>Arancio/Orange tree	Fruits/infructescences/accessory fruits (dandelion flowers in infusion with lemon, orange, and raisin)	Med	DIG	Digestive			
Ruta graveolens L.<br>Ruta/Rue	Underground organs (decoction)	Med	GC	Anti-inflammatory		Anti-inflammatory [227,228]	Anti-inflammatory: phenolic compounds (rutin, syringic acid, and naringenin) [227]<br>Anti-inflammatory: quinoline alkaloids (skimmianine) [228]
**Scrophulariaceae**							
Buddleja davidii Franch.<br>Albero delle farfalle/Butterfly bush<br><br>Probably mistaken for *Vitex agnus-castus* L.	Unknowkn (Leaves or flowers) (infusion)	Med	OTH	Lowers male hormones			
Verbascum thapsus L.<br>Tasso barbasso/ Great mullein	Leaves (infusion)	Med	RES	Cough	[33,36,58–60]	Expectorant, mucolytic [229]	Effects on respiratory system: iridoid glycosides (catalpol, aucubin, saccatoside, harpagoside, harpagide); flavonoids

							(apigenin, luteolin, rutin, quercetin, kaempferol, cynaroside); polysaccharides, steroids and sterones, lignans, sesquiterpenes, terpenoid saponins and phenolic compounds (vanillic acid), phenylethanoid glycosides (verbascoside) [229]
**Solanaceae**							
Solanum tuberosum L. Patata/Potato	Underground organs (applied as they are, raw)	Med	SK	Sunburn, burns, frostbites, redness, and rash	[33,35]	Anti-inflammatory [230–232]	Anti-inflammatory: glycoalkaloids (α-chconine, α-solanine, solanidine) [230,231] Anti-inflammatory: isolated peptides [233] Antioxidant: chlorogenic acid, proteins (patatins), peptides [231]
	Underground organs (applied as they are, raw)	Med	OPT	Eye anti-inflammatory	[33,34,36,45]	Anti-inflammatory [230–232]	
**Urticaceae**							
Urtica dioica L. Ortica/Nettle	Leaves (infusion)	Med	SK	Against hair loss, strengthens the scalp	[32,37,58,60]	[233]	
	Leaves (infusion)	Med	UR	Diuretic	[33,36,37,59,60,90]		
	Leaves (external application)	Med	CS	Improves circulation	[46]		
	Leaves (eaten in soups)	Med	RES	Bronchitis	[33]		

	Leaves (eaten cooked)	Med	GC	Tonic and corroborant	[33,34,36,46]	Rich in vitamins and minerals [233]	
	Leaves (infusion)	Med	GYN	Anti-inflammatory and soothing, redness Emmenagogue, regulates menstrual cycle	[36]	Anti-inflammatory [234]	
	Above ground parts (external application)	Med	MSK	Joint pain and inflammation	[33,34,36,37,45,46,58]	Anti-inflammatory [234]	
**Verbenaceae**							
Aloysia citrodora Paláu<br>Verbena odorosa/Lemon verbena	Leaves (infusion)	Med	GC	Anti-inflammatory		[235]	Anti-inflammatory: citral [235]
**Viburnaceae**							
Sambucus nigra L.<br>Sambuco nero/Black elder	Flowers/inflorescences/flowering tops (infusion)	Med	DIG	Laxative, bowel transit	[33,34,36,58,90]	[236]	Anthocyanins (Cyanidin-3-sambubioside, Chrysanthemin, Cyanidin-3,5-diglucoside); Proanthocyanidins (epicatechin); Flavonoids (Rutin, isoquercetin); Peptic polysaccharides; Lectins (SNA-IV, SNA-V) of berries extracts: immunostimulant, antiviral (against flu), and antimicrobic activities [237]
	Flowers/inflorescences/flowering tops (infusion)	Med	NS	Sedative hypnotic, promotes sleep	[35]		
	Flowers/inflorescences/flowering tops (infusion, syrup)	Med	RES	Expectorant, decongestant, emollient Sore throat and hoarseness Cold Cough	[32–37,44–46]	[237–240]	

	Fruits/infructescences/accessory fruits (syrup)	Med	RES	Cough	[32–37,44–46]	[237–240]
	Flowers/inflorescences/flowering tops (infusion)	Med	FE	FE and infections	[35]	[240]
	Fruits/infructescences/accessory fruits (syrup)	Med	DIG	Laxative, bowel transit	[33,34,36,58,90]	[236]
	Rhytidome (compresses with infusion)	Med	SK	Anti-inflammatory, disinfectant, wound healing	[35,44–46]	Anti-inflammatory [241]
**Vitaceae**						
Vitis apyrena L. Uvetta/Raisin	Fruits/infructescences/accessory fruits (infusion) With dandelion flowers, lemon, and orange juice	Med	DIG	Digestive		
Vitis labrusca L. Vite americana/Fox grape	Fruits/infructescences/accessory fruits (eaten as it is)	Med	GC	Tonic and corroborant		
Vitis vinifera L. Vite, uva/Grapes	Pomace (grappa, mouthwashes)	Med	OR	Gingivitis, toothache, aphthae, and abscesses	[46]	
	Leaves	Med	MSK	Joint pain and inflammation		

	(applied as they are)					
**Xanthorrhoeaceae**						
Aloe vera (L.) Burm.f. Aloe/Aloe	Leaves, gel	Med	GC	Tonic and corroborant	Immunomodulant [242]	Polysaccharides [242]
**Zingiberaceae**						
Zingiber officinale Roscoe Zenzero/Ginger	Underground organs (infusion)	Med	RES	Sore throat and hoarseness Facilitates respiration	[243]	Anti-inflammatory: 6-shogaol and 6-gingerol, 6-dehydroshogaol, zingerone [243] Gastroprotective: zingerone [244]
	Underground organs (infusion)	Med	DIG	Antispasmodic (stomach) Digestive	Gastroprotective and antiemetic [243–245]	
	Underground organs (decoction)	Med	GC	Anti-inflammatory	[243]	
	Underground organs (decoction)	Med	GC	Depurative		
